# Diversity of fungal feruloyl esterases: updated phylogenetic classification, properties, and industrial applications

**DOI:** 10.1186/s13068-016-0651-6

**Published:** 2016-10-28

**Authors:** Adiphol Dilokpimol, Miia R. Mäkelä, Maria Victoria Aguilar-Pontes, Isabelle Benoit-Gelber, Kristiina S. Hildén, Ronald P. de Vries

**Affiliations:** 1 Fungal Physiology, CBS-KNAW Fungal Biodiversity Centre & Fungal Molecular Physiology, Utrecht University, Uppsalalaan 8, 3584CT Utrecht, The Netherlands; 2 Division of Microbiology and Biotechnology, Department of Food and Environmental Sciences, University of Helsinki, P.O. Box 56, 00014 Helsinki, Finland

**Keywords:** Feruloyl esterase, Ferulic acid, Cinnamic acid, *P*-coumaric acid, Hydroxycinnamic acid, Plant cell wall, Phylogenetic analysis, Applications, Biotechnology

## Abstract

Feruloyl esterases (FAEs) represent a diverse group of carboxyl esterases that specifically catalyze the hydrolysis of ester bonds between ferulic (hydroxycinnamic) acid and plant cell wall polysaccharides. Therefore, FAEs act as accessory enzymes to assist xylanolytic and pectinolytic enzymes in gaining access to their site of action during biomass conversion. Their ability to release ferulic acid and other hydroxycinnamic acids from plant biomass makes FAEs potential biocatalysts in a wide variety of applications such as in biofuel, food and feed, pulp and paper, cosmetics, and pharmaceutical industries. This review provides an updated overview of the knowledge on fungal FAEs, in particular describing their role in plant biomass degradation, diversity of their biochemical properties and substrate specificities, their regulation and conditions needed for their induction. Furthermore, the discovery of new FAEs using genome mining and phylogenetic analysis of current publicly accessible fungal genomes will also be presented. This has led to a new subfamily classification of fungal FAEs that takes into account both phylogeny and substrate specificity.

**Electronic supplementary material:**

The online version of this article (doi:10.1186/s13068-016-0651-6) contains supplementary material, which is available to authorized users.

## Background

Plant biomass is a magnificent renewable source of biopolymers. It offers a wealth of possibilities for development and production of sustainable raw materials and energy which fit perfectly with the development of a bio-based economy [1]. Lignocellulosic biomasses from agricultural, agro-industrial, crop, and forestry wastes as well as herbaceous prairie grass, energy crops, and marine algae are regarded as the prospective feedstocks for modern bioethanol and biochemical production [2, 3]. The enzymatic hydrolysis of lignocellulosic biomass has many advantages when compared to chemical conversion in bioethanol production. There is no substrate loss due to chemical modifications; non-corrosive operational conditions may be used and the process is more environmentally friendly [4]. However, plant cell walls have evolved to defend against external factors, including mechanical, thermal, chemical, and biological stress [5, 6]. To efficiently and completely depolymerize different types of lignocellulosic materials, an arsenal of carbohydrate-active and lignin-acting enzymes is required [7, 8]. Feruloyl esterases (FAEs, also known as ferulic/cinnamic acid esterases, EC 3.1.1.73) are responsible for removing ferulic acid residues and cross-links from polysaccharides. They act as accessory (or auxiliary) enzymes that assist the other enzymes in gaining access to their site of action during biomass conversion [9, 10]. In addition to their potential role in bioethanol production, FAEs and their hydrolytic or transesterification products are of great interest for various biotechnological applications, in particular as modified natural antioxidants or food flavor precursors [11, 12]. Therefore, discovery of new FAEs with novel properties and applications is of considerable interest to industry [13]. In this review, we describe (1) the roles of FAEs in plant biomass degradation, (2) an overview of biochemical properties as well as the conditions that induce FAE production, (3) discovery of FAEs and insight into their phylogenetic relationships among fungal genomes, (4) an updated subfamily classification for fungal FAEs and (5) the recent applications of FAEs in biotechnological processes.

### Ferulic and hydroxycinnamic acids in plant cell walls

**Fig. 1:**
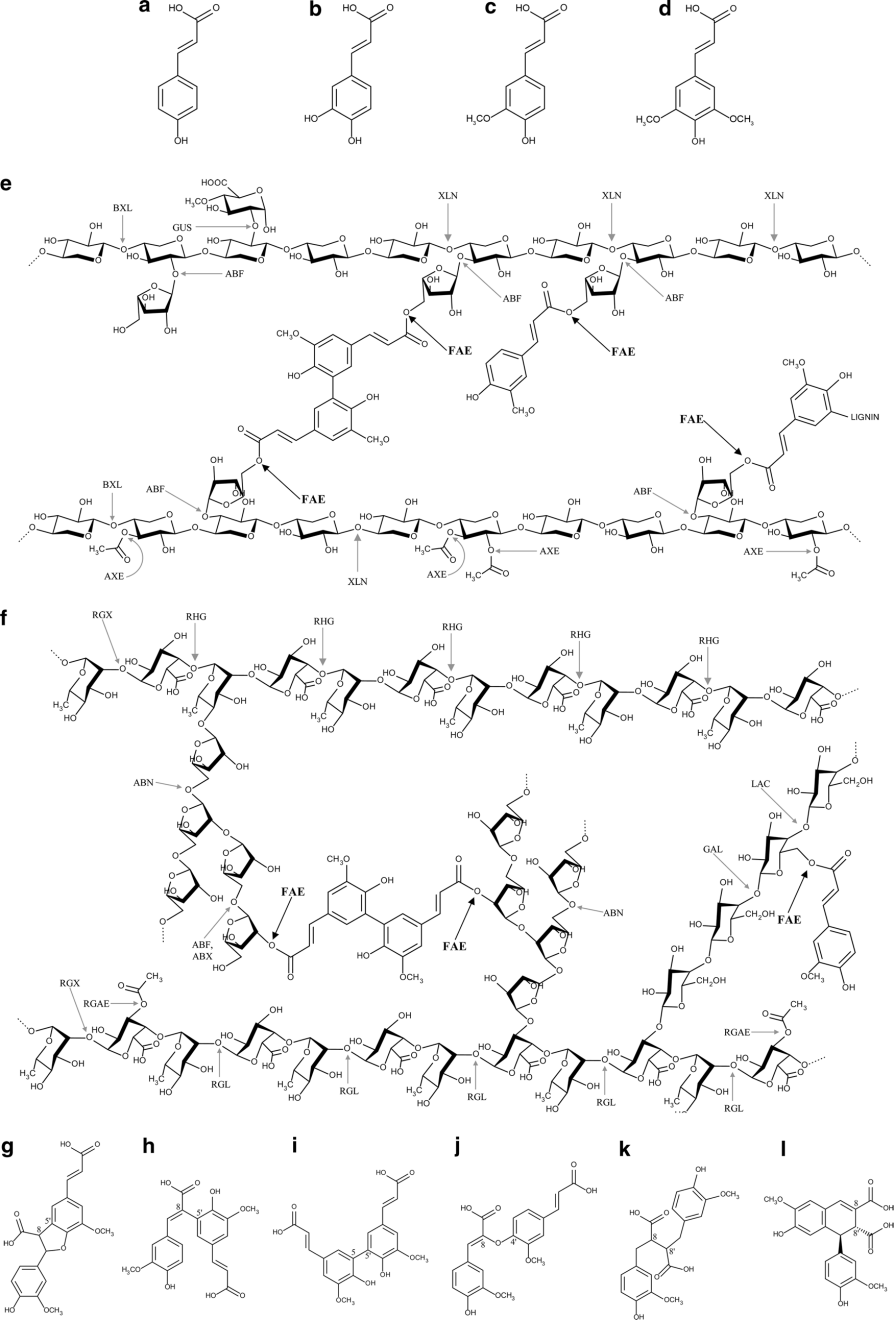
Model structures of hydroxycinnamic acids, feruloylated plant cell wall polysaccharides and the site of attack by the carbohydrate-active enzymes (modified from [8, 15]). **a** *p*-coumaric acid, **b** caffeic acid, **c** ferulic acid, **d** sinapic acid, **e** feruloylated glucuronoarabinoxylan, **f** feruloylated pectic rhamnogalacturonan I, **g** 8,5′-(benzofuran)-diferulic acid, **h** 8,5′-diferulic acid, **i** 5,5′-diferulic acid, **j** 8,4′-diferulic acid, **k** 8,8′-diferulic acid, **l** 8,8′-(aryl)-diferulic acid. *ABF* α-arabinofuranosidase; *ABN* endoarabinanase; *ABX* exoarabinanase; *AXE* acetyl xylan esterase; *BXL* β-1,4-xylosidase; *FAE* feruloyl esterase; *GAL* β-1,4-endogalactanase; *GUS* α-glucuronidase; *LAC* β-1,4-galactosidase; *RGAE* rhamnogalacturonan acetyl esterase; *RGL* rhamnogalacturonan lyase; *RGX* exorhamnogalacturonase; *RHG* endorhamnogalacturonase; *XLN* β-1,4-endoxylanase

Different types of lignocellulosic biomass can be used for second generation bioethanol production. In order to select the appropriate specificity of enzymes required for biomass degradation, we briefly summarize the occurrence of ferulic and hydroxycinnamic acids in the different types of plant biomass. A variety of hydroxycinnamic acids are present in the plant cell walls (up to 3% of cell wall dry weight), usually esterified or etherified to the polymers within the lignocellulosic matrix [14, 15]. Ferulic acid (ferulate, 4-hydroxy-3-methoxycinnamic acid, mainly *trans*- or *E*-form; Fig. 1) and to a lesser extent *p*-coumaric acid (*p*-coumarate, 4-hydroxycinnamic acid) are the most abundant hydroxycinnamic acids (hydroxycinnamates) in the plant cell wall polysaccharides. They are regarded as essential and unique structural components in the family Poales of commelinid monocots (e.g., wheat, rice, barley, oat, corn, sorghum, and sugarcane) [15, 16]. Ferulic acid is linked to cell wall polysaccharides mainly through ester-bonds between their carboxyl group and the *O*-5 hydroxyl group of α-l-arabinofuranosyl residues in glucuronoarabinoxylan (Fig. 1e) [17, 18]. In eudicotyledons, ferulic acid is mainly found in the order ‘core’ Caryophyllales (e.g., sugar beet; [19]). It is ester-linked to pectin at the *O*-2 and *O*-5 hydroxyl group of α-l-arabinofuranosyl residues in arabinan as well as at the *O*-6 hydroxyl group of β-d-galactopyranosyl residue in (arabino-)galactan, both of which are side chains of rhamnogalacturonan I (Fig. 1f) [16, 20–22]. Ferulic acid can oxidatively cross-link to form intermolecular ester-bonds to another arabinoxylan [mainly 5,5′-, 8-*O*-4′-, 8,5′-, 8,8′-diferulic acids (Fig. 1 g–l)], and ester-ether bonds between polysaccharide and lignin (arabinoxylan–ferulate–lignin) [15, 16, 19, 23–25]. Diferulic acids have been mainly detected in the high-arabinose substitution region of arabinoxylan, because dimerization requires the ferulic acid to be in close proximity [26]. In addition, cross-linking of cell wall polysaccharides and lignin by hydroxycinnamic acids leads to a dramatic increase in mechanical strength of the plant cell wall, decelerates wall extension, and acts as a barrier to block the ingress of microbial invaders as well as hydrolytic enzymes [16, 23, 27].

Ferulic acid is also detected in all families of gymnosperms, ester-linked to the primary cell walls, with an amount ranging from 0.01 to 0.16% [28]. However, up to now there is still no identification of which polysaccharides ferulic acid is linked to in gymnosperms [14].

### Role of FAEs in plant biomass degradation

Opening up the plant cell wall is a significant part of the process design for bioethanol and biochemical production. Due to the heterogeneity and complexity of the plant cell walls, a variety of carbohydrate- and lignin-active enzyme sets with complementary activities and specificities are required for complete enzymatic hydrolysis of plant biomass (for details see [8, 29]). As ferulic acid is linked to the lignin–carbohydrate complexes, disruption of the ester bond of the lignin–ferulate–arabinoxylan complex is important for complete cell wall deconstruction. FAEs play a key role in providing accessibility for glycoside hydrolases and polysaccharide lyases to the lignocellulose fibers by removal of the ester-bonds between plant polymers [9, 10]. FAEs not only act synergistically with xylanolytic enzymes to convert xylan into its monomers, but have also proved to enhance overall saccharification of lignocellulosic biomass, e.g., wheat straw [30] and sugarcane bagasse [31], when co-incubated with cellulase and xylanase. Moreover, overexpression of FAEs in planta reduces the levels of cell wall esterified phenolics and in most cases also enhances sugar release and improves cell wall digestibility [32–36]. This technique has also been applied to create self-processing transgenic plants that can alter their composition upon activation of the enzyme(s), e.g., to reduce recalcitrance of cell walls prior to saccharification (e.g., [37]; see application section below).

## Overview of substrate specificity of characterized FAEs and their other properties

### FAE discovery

The first FAEs were discovered in the late 80’s when a new type of esterase capable of releasing the covalently linked ferulic acid from xylan was reported [38–41]. During this period, most FAEs were identified by direct purification from culture supernatant, which required an appropriate induction condition [42–44]. The first fungal FAE encoding genes were identified from *Aspergillus niger* and *Aspergillus tubingensis* [45]. Peptide sequencing was used to identify short amino acid sequences of FAEs followed by degenerate or rapid amplification of cDNA ends PCR to obtain the whole gene sequence (Additional file 1: Table S1). Screening of cDNA libraries was also used for discovery of FAEs particularly for anaerobic rumen fungi (e.g., [46–48]). In recent years, publicly available fungal genome sequences have facilitated similarity-based discovery, and genome mining has become the most promising discovery technique (Additional file 1: Table S1). Databases such as carbohydrate-active Enzymes (CAZy) database (http://www.cazy.org; [49]) are very powerful tools for discovery of alternative enzymes of existing families. Discovery of novel enzyme classes or alternative enzymes from enzyme families not included in the database (such as several FAE families) requires other approaches. However, it should be noted that FAEs are a very diverse enzymes, so similarity-based discovery does not necessarily guarantee the same function.

### Activity and properties of FAEs

Although FAEs have been identified in various plant cell wall degrading microbes, to date fungi are still the main source of FAEs used in industry [50]. Thorough data collection for the physicochemical properties of purified FAEs has been previously reported [9, 50–53], and therefore here we only present the properties of characterized fungal FAEs for which amino acid sequences have been reported (Additional file 1: Table S1; see also update classification section).

FAEs are active in a broad pH (from pH 3 to 10) and temperature (from 20 to 75 °C) range, but generally they are mainly active at pH 4–7 and temperatures below 50 °C (Additional file 1: Table S1; [50, 53]). A few reports also showed the effect of metal ions and inhibitors on FAEs [54–57]. It should be noted that the structures of only two fungal FAEs have been reported until now: *A. niger* (AnFaeA—[58–61]) and *Aspergillus oryzae* (AoFaeB—[62]), of which only AoFaeB contains a calcium binding site in its structure. It is located far from the active site but may have a role in stabilizing the protein structure. FAEs catalyze the hydrolysis of the substrate following the mechanism utilized by serine proteases [63] with a conserved Ser-His-Asp/Glu catalytic triad [64]. Glu as a part of catalytic triad, instead of Asp, was recently reported in several Basidiomycetes, which is uncommon among FAEs, but found in some members of the α/β-hydrolase-fold superfamily [57, 65, 66]. Differences in amino acid residues within loops and domains that situate in close proximity to the catalytic and substrate binding sites enable different FAEs to target different substrates [59, 62, 64]. The catalytic mechanism of FAEs involves two steps, the initial acylation of the nucleophilic serine residue forming acyl-enzyme intermediate followed by deacylation of the intermediate. In the deacylation step, nucleophilic water (hydrolysis) or other hydroxyl molecule from e.g., carbohydrate or alcohol (transesterification, see also industrial applications section) can attack the intermediate and cause the release of the product [58, 64].

Different substrates were used for characterization of FAEs: polysaccharides (e.g., wheat bran and sugar beet pulp [67]), feruloylated oligosaccharides (e.g., feruloylate-Ara-Xyl_1–3_, feruloylate-Ara_1–3_, *p*-coumaroylate-Ara-Xyl_1–3_ [22]), and monomeric hydroxycinnamate model substrates (e.g., methyl, ethyl, *p*-nitrophenyl, or α-naphthyl ferulate [46, 68–70]). Short chain fatty acid model substrates (e.g., α-naphthyl acetate, umbelliferyl acetate, and umbelliferyl butyrate) are also used for the activity assay. However, they only show whether the enzyme is active, but not whether they are specific to ferulic or hydroxycinnamic acid.

Reversed phase HPLC/UV is the most used technique for detection of ferulic and hydroxycinnamic acids, and their release from feruloylated poly- and oligosaccharides [22, 71, 72]. However, it is time consuming and usually requires prior isolation/extraction step before the analysis, which makes it less useful for rapid or high-throughput screening [70]. For the activity screening, a spectrophotometry-based method using monomeric hydroxycinnamate model substrates which detects the release of chromophore group (e.g., *p*-nitrophenyl, α-naphthyl ferulate) or the reduction of substrate (e.g., methyl, ethyl ferulate) is rapid and easy to perform. The spectrophotometric assay is widely accepted, even though there is a concern about the spectral overlapping between substrate and product, e.g., methyl substrates and their aromatic acids. Recently, other methods have been developed such as high-performance thin layer chromatography and electrochemical sensor for rapid detection of ferulic acid which may be useful for enzymatic screening [73, 74].

Several fungi produce more than one FAE isozyme and different substrates are required to determine their substrate specificity. The classical examples are two *A. niger* FAEs, AnFaeA and AnFaeB [45, 67, 75]. Regarding the monomeric substrates, AnFaeA is specific for ferulic and sinapic acid methyl esters, while AnFaeB is specific for ferulic, *p*-coumaric, and caffeic acid methyl esters (Fig. 1a–d). Of the oligomeric substrates (derived from wheat bran and sugar beet pulp), AnFaeA catalyzes the hydrolysis of the feruloylated (1,5) arabinosyl *xylo*-oligosaccharides from wheat arabinoxylan, but is less active towards feruloylated (1,2) arabino- and (1,6) galacto-oligosaccharides from sugar beet pulp. AnFaeB is active towards feruloylated oligosaccharides derived from both monocot and dicot cell walls [15, 68, 76]. Regarding the polymeric substrates, both FAEs also show opposite substrate preference. AnFaeA highly prefers to hydrolyze wheat arabinoxylan over sugar beet pectin and can also release the diferulic acid (5,5′, 8-*O*-4′), whereas AnFaeB is more active towards sugar beet pectin but cannot release diferulic acid [15, 75].

### Inducing substrates, regulation, and production

Production of FAEs in nature depends highly on the available carbon sources or inducing compounds. Ferulic acid, and related hydroxycinnamic acids (e.g., caffeic, *p*-coumaric acids) and phenolic compounds (e.g., vanillic acid, vanillin, and veratric acid) can induce the production of FAEs [75]. Feruloylated plant biomass such as wheat bran, sugar beet pulp/pectin, and maize bran are frequently used as substrates for production of FAEs (Additional file 1: Table S1). Recent transcriptomic data from different fungal species suggested that the low- to non-feruloylated biofuel feedstocks such as the straw from wheat, barley, corn, rice, and soybean as well as the woody substrates from both softwood (pine) and hardwood (aspen) can substantially upregulate the expression of *fae* genes [77–80]. Although the presence of ferulic acid in the cultivation is not absolutely required, addition of ferulic acid can considerably improve the production of FAEs [76]. Xylose induces the production of AnFaeA, whereas most monosaccharides do not appear to support the production of other FAEs [75].

Detailed expression studies of FAE encoding genes are rare and have so far been mainly performed in species of the genus *Aspergillus*. Here expression of *fae* genes is presumed to be controlled by at least three independent regulatory systems [81]. The xylanolytic transcriptional activator XlnR, a zinc binuclear cluster motif (Zn(II)_2_Cys_6_), is a key factor in the regulation of hemicellulolytic and cellulolytic genes in Aspergilli [82–84]. In *A. niger*, *faeA* and other genes encoding xylan degrading enzymes (e.g., *xlnB*, *xlnC*, *xlnD*, *axeA*, *axhA*, and *aguA*) are under control of XlnR [81, 82]. Another major regulator that is responsible for carbon catabolite repression in many filamentous fungi is the conserved zinc-finger regulator CreA [85, 86]. Expression of *faeA* was influenced by the balance between induction by XlnR and repression by CreA, whereas *faeB* was not activated by XlnR, but still sensitive to CreA-mediated repression [75, 87]. *creA* deletion mutants showed improved production of secreted lignocellulose degrading enzymes including FAEs [75, 77]. In addition, both *faeA* and *faeB* are expressed in the presence of ferulic acid and other hydroxycinnamic acids [75], indicating the presence of a ferulic acid- or hydroxycinnamic acid-responsive transcriptional regulator. It should be noted that the ferulic acid induction is independent of XlnR and the combined ferulic acid induction and XlnR effect on expression of *A. niger faeA* is larger than the sum of the two effects alone [81]. However, it is unclear whether the ferulic acid- or hydroxycinnamic acid induction is mediated by a single regulatory system since different sets of phenolic compounds induced the expression of *faeA* and *faeB* [75, 88]. As FaeA is only found in Aspergilli and related species (see below), it is currently unclear to which extent XlnR orthologs in other fungi are involved in activating expression of FAE encoding genes.

Native fungal FAEs are produced mainly through two types of cultivation techniques: submerged/liquid fermentation in which fungi are grown in liquid medium often with vigorous aeration; and solid-state fermentation in which they grow on moist solid substrates such as lignocellulosic biomass. Although FAE production from native sources can reach high levels, e.g., >10^6^ mU/mL for submerged fermentation of *Aspergillus awamori* [54] and >10^3^ mU/g for solid-state fermentation of *Penicillium brasilianum* [89], production of FAEs from native sources faces considerable complications e.g., the choices of suitable substrates, control of fermentation conditions, up-scaling and the purification process [90]. Over the past decade, FAE production has shifted more towards heterologous mainly using two expression hosts, i.e., *Escherichia coli* and *Pichia pastoris* under the isopropyl β-d-1-thiogalactopyranoside (IPTG) or methanol inducible promoters, respectively, for Academia (Additional file 1: Table S1). For industry, the established platforms of the company are being used. Heterologous production offers several advantages over native production, such as well-established cultivation conditions for up-scaling, fusion of affinity tags for downstream processing and possibilities for enzyme engineering.

### Classification of FAEs

The initial classification of FAEs was based on the induction and substrate specificity of AnFaeA and AnFaeB [15, 91]. Subsequently, based on the substrate specificity towards four model substrates (methyl ferulate, sinapate, caffeate, and *p*-coumarate) and the ability to release diferulic acid, FAEs were classified into four types (A, B, C and D) [92]. Type A FAEs prefer substrates containing methoxy substitutions at C-3 and/or C-5 as found in ferulic and sinapic acids, and are active towards methyl *p*-coumarate. They are also capable of releasing 5,5′- and 8-*O*-4′-diferulic acids. Type B FAEs prefer substrates containing one or two hydroxyl substitutions, as found in *p*-coumaric and caffeic acids, respectively. Hydrolytic rates of type B FAEs are significantly reduced when a methoxy group is present and they are not active against methyl sinapate. In addition, type B FAEs cannot release diferulic acid. Type C and D FAEs possess broader substrate specificity with activity towards all four model substrates, but only type D can release diferulic acid from plant cell walls [92].

The ABCD classification was very useful and initially was supported by phylogenetic analysis because a limited number of amino acid sequences of FAEs were available at that time. As more FAEs were characterized, it no longer adequately reflects the wealth of putative FAEs encoded by microbial/fungal genomes. Hence, a refined classification was introduced based on phylogenetic analysis of available fungal genomes, which separated FAEs into seven subfamilies (SF1-7) [51]. This classification demonstrated that FAEs evolved from highly divergent esterase families (tannases (SF1-4), acetyl xylan esterases (SF6), and lipases (SF7)) and do not have a common ancestor, even though they all contain a conserved Ser-His-Asp catalytic triad [51]. The availability of fungal genome sequences also enabled a more detailed comparison of the diversity and prevalence of putative FAEs [93, 94]. Although FAEs are carbohydrate-active enzymes, they are only partially included in CAZy database [49] as some FAEs (SF5, SF6) belong to carbohydrate esterase family 1 (CE1) together with acetyl xylan esterases. More recently, a further refined classification was proposed by clustering 365 FAE-related amino acid sequences using descriptor-based computational analysis and machine learning algorithms [52]. At the same time, pharmacophore models for specific FAE subgroups were also developed, which will be useful for production of FAE-based biosynthetic compounds. The descriptor-based classification separated the FAEs into 12 families; however, some of these families were further divided into subgroups (A–D) to distinguish the substrate specificity of characterized FAEs within the family.

## Update on the classification of fungal FAEs

### New phylogenetic tree based on all published fungal genomes

Based on the previously reported phylogenetic analysis [51], we reconstructed a novel phylogenetic tree using 20 amino acid sequences from characterized FAEs (Table 1) and a BLASTP search against 247 published fungal genomes (Additional file 1: Table S2). All resulting amino acid sequences with an expect value lower than 1E^−40^ were collected. Duplicate and incomplete sequences as well as sequences with ambiguous amino acids (X) were discarded. Signal peptides were predicted using SignalP [95] and removed from all candidate sequences. This analysis resulted in 1251 putative FAE sequences, which were aligned using Multiple Sequence Comparison by Log-Expectation (MUSCLE). Phylogenetic analysis was performed using the neighbor-joining method with pairwise deletion of gaps and the Poisson correction distance of substitution rates (statistical support for phylogenetic grouping was estimated by 1000 bootstrap re-samplings) of the Molecular Evolutionary Genetics Analysis (MEGA 6) program [96]. A few characterized acetyl xylan esterases, lipases, and tannases were included in the analysis to reveal the relationships of FAEs with those enzymes. In this analysis, the xylanase-related FAEs (e.g., FAEs from *Aspergillus terreus* (AtFAE-2, AtFAE-3) [56]) were not included in the similarity search because they showed similarity to GH10 and GH11 xylanases. Including these xylanase-related FAEs also recognized other non-FAE members of these two families, which could not be screened out because only two fungal xylanase-related FAEs were identified. Also, a putative FAE from *Xylaria polymorpha* (XpoGH78) [97] was not included in the phylogenetic tree because it showed no relationship with other FAE sequences.

**Table 1:** Properties and classification of fungal FAEs with reported amino acid sequences

Origin	Enzyme	Apparent	pH		Temp (°C)		pI	Type	FEF	SF	New	Accession number	References
		Mass (kDa)	Optimum	Stability	Optimum	Stability					SF		
								(A–D)	(1–12)	(1–7)	(1–13)		
*Aspergillus nidulans*	AN1772	130	7.0	4.0–9.5	45	<40	–	B,c	4A	1	1	XM654284	[159]
*Aspergillus niger* ^a^	AnFaeB/CinnAE	75	6.0	–	–	–	4.8	B,c	4A	1	1	Q8WZI8.1	[75]
*Aspergillus oryzae*	AoFaeB	61	6.0	3.0–9.0	–	<55	–	B,c	12B	1	1	XP_001818628	[98]
*Aspergillus oryzae*	AoFaeC	75	6.0	7.0–10.0	–	<55	–	C	4B	1	1	XP_001819091	[98]
*Penicillium chrysogenum* ^a^	PcFAEI	62	6.0–7.0	4.0–7.0	50	<55	–	B,c	4A	1	1	BAE44304	[160]
*Talaromyces stipitatus*	TsFAEC	66	6.0–7.0	4.0–7.0	60	<60	4.6	C	4B	1	1	CAD44531.1	[161]
*Fusarium oxysporum* ^a^	FoFaeC	62	6.0	4.0–10.0	65	<40	6.8	C	4B	–	2	FOXG_12213	[103]
*Aspergillus clavatus*	AcFAE	30	7.0	6.0–8.5	30	–	–	D	–	5	5	XP_001274884	[162]
*Aspergillus nidulans* ^a^	AnidFAE/AN5267	28	–	–	–	–	–	–	–	5	5	EAA62427.1	[99]
*Myceliophthora thermophila* ^a^ *(Chrysosporium lucknowense)*	ClFaeA1	29	7.0	–	45	–	5.5	A	–	5	5	AEP33616.1	[163]
	ClFaeA2	36	7.5	–	40	–	5.2	A	–	5	5	AEP33617.1	
*Neurospora crassa* ^a^	NcFaeD	32	–	–	–	–	–	D	4D	5	5	XP_956228	[91]
*Talaromyces funiculosus* ^a^ *(Penicillium funiculosum)*	PfFaeA	–	–	–	–	–	–	D	–	5	5	AJ312296	[164]
*Chaetomium sp.CQ31* ^a^	ChaeFae	30	7.5	4.0–10	60	<55	–	–	–	6	6	AFU88756.1	[100]
*Myceliophthora thermophila*	MtFae1a	39	7.0	7.0–10.0	50	<55	–	B	–	–	6	AEO62008.1	[165]
*Myceliophthora thermophila*	ClFaeB2	33	7.0		45	–	6	B	–	6	6	AEP33618.1	[163]
*Neurospora crassa* ^a^	NcFae1	35	6.0	6.0–7.5	55	–	–	B	6A	6	6	CAC05587.1	[166]
*Talaromyces funiculosus* ^a^ *(Penicillium funiculosum)*	FaeB	53	–	–	–	–	6	B	5B	6	6	CAC14144	[69]
*Aspergillus awamori*	AwFaeA	35	5.5	4.0–8.0	55	25–75	4.2	A	12A	7	7	BAA92937.3	[167, 168]
*Aspergillus flavus* ^a^	AfFaeA	40	6.0	4.5–8.0	58	40	–	A	–	–	7	AGN75069.1	[101]
*Aspergillus niger* ^a^	AnFaeA/FAE-III	36	5.0	–	60	–	3.3	A	12A	7	7	CAA70510	[45]
*Aspergillus oryzae*	AoFaeA	37	5.0	4.0–6.0	50	<52	–	A	–	7	7	AHZ18111.1	[102]
*Aspergillus terreus*	AtFAE-1	76	5.0	3.0–8.0	50	<50	–	A	12A	7	7	*Sim:* EAU31039.1	[56]
*Aspergillus tubingensis*	AtubFaeA	30	–	–	–	–	–	–	12A	7	7	CAA70511	[45]
*Aspergillus usami*	AuFaeA	36	5.0	4.0–6.5	45	<45	4.3	A	–	7	7	AHB63528.1	[169]
*Anaeromyces mucronatus* ^a^	Fae1A	37	7.2	5.5–8.0	37	<15	–	A	–	–	8	ADZ47894.1	[48]
*Auricularia auricula*-*judae* ^a^	EstBC	36	6.5	3.5–8.0	61	<65	3.2	–	–	–	8	*Sim*: EJD51015	[104]
*Orpinomyces sp.* ^a^	FaeA	–	–	–	–	–	–	–	1A	–	8	AAF70241.1	[105]
*Pleurotus eryngii* ^d^	PeFaeA	67	5.0	–	50	<50	–	A	–	–	12	CDI44666	[57]
*Pleurotus sapidus*	Est1	55	6.0	–	50	–	–	A	–	–	12	CBE71381	[106]
*Ustilago maydis*	UmChlE	71	7.5	3.5–9.5	37	<40	3	B	–	–	13	HG970190	[65]
*Coprinopsis cinerea* ^a^	CcEst1	46	–	–	–	–	–	–	–	–	U8	BAJ10857.1	[111]
*Piromyces equi* ^a^	PeEstA	55	6.5	6.0–8.0	50–60	<50	–	D	2	–	U8	AAD45376.1	[46, 110]
*Piromyces *sp.	FaeA	–	–	–	–	–	–	–	–	–	U7	AAP30751	[47]
*Aspergillus terreus*	AtFAE-2	23	5.0	3.0–8.0	40	<40	–	C	–	–	U5	*Sim:* EAU39455.1	[56]
*Aspergillus terreus*	AtFAE-3	36	5.0	3.0–8.0	40	<40	–	C	–	–	U2	*Sim:* XP_001214121.1	[56]
*Xylaria polymorpha*	XpoGH78	98	6.0–8.0	–	45	<40	3.7	–	–	–	N^3^	AFA53086.1	[97]

*B*,*c* substrate specificity profiling of type B, but high sequence similarity to type C; *Sim* the peptide sequences are similar to; *N* not included in the phylogenetic analysis

^a^Indicates the amino acid sequences used for genome mining of fungal FAEs

**Fig. 2:**
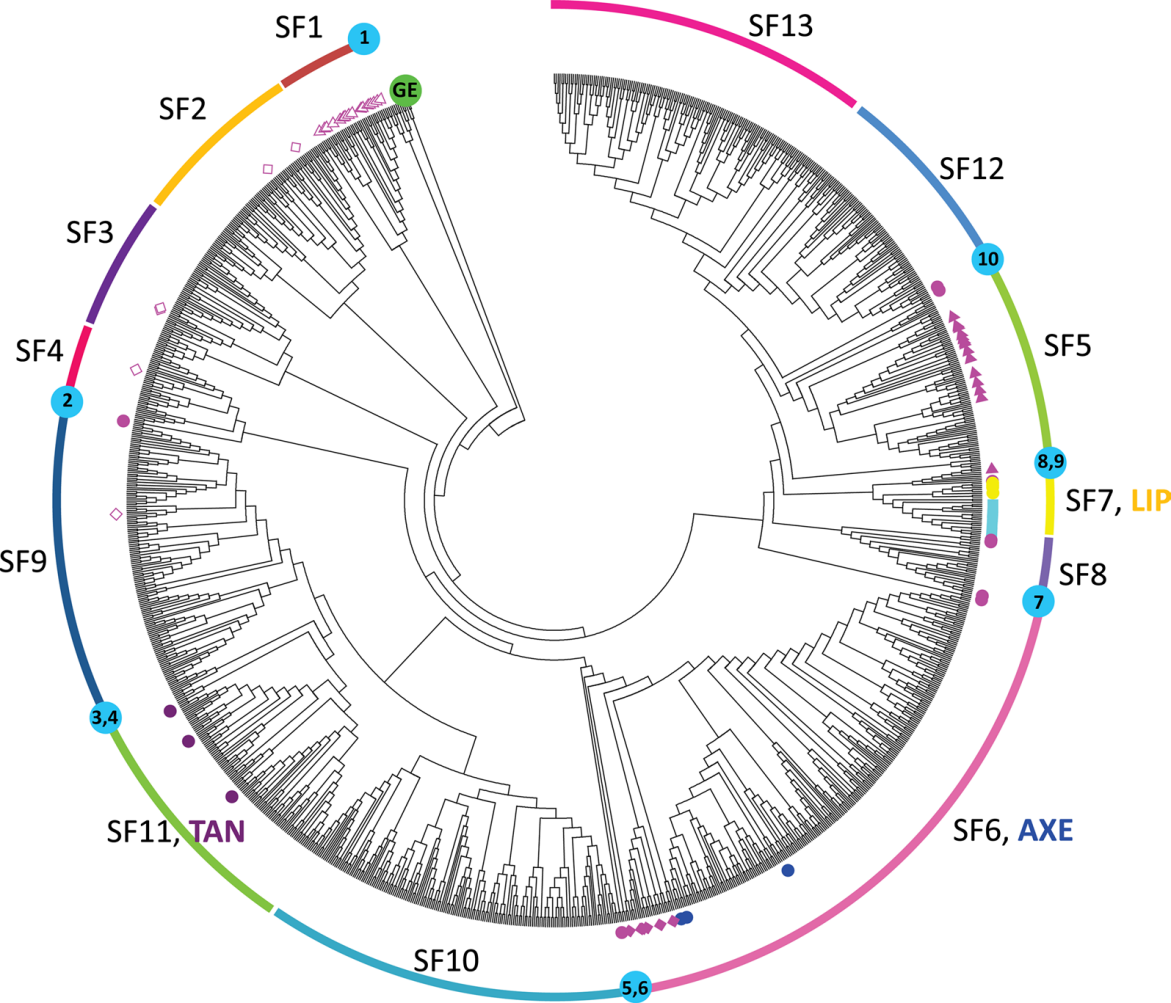
Phylogenetic relationships among the (putative) fungal FAEs. Glucuronoyl esterases (GEs, *green-filled circles*) were used as an outgroup. AXE, acetyl xylan esterase (*blue-filled circles*); LIP, lipase (*yellow-filled squares*); SF, subfamily; TAN, tannase (*purple-filled circles*). FAEs from previously reported phylogenetic analysis [51] were marked with *magenta open triangles* for SF1, *magenta open rhombuses* for SF2-4, *magenta-filled triangles* for SF5, *magenta-filled rhombuses* for SF6, *light blue-filled squares* for SF7, *brown-filled circles* for AtFAE2 and AtFAE3, and *magenta-filled circles* for ungrouped ones. *Light* *blue-filled circles* indicate ungrouped sequences which numbering indicates different groups

Previously, the phylogenetic analysis classified the FAEs into seven subfamilies [51] (Table 1; Additional file 1: Table S1). SF1 contained FAEs from *A. niger* (AnFaeB [75]) and *A. oryzae* (AoFaeB, AoFaeC [98]) which are closely related to tannases. SF5 contained FAEs from *A. nidulans* (AN5267 [99]) and *Neurospora crassa* (NcFaeD [91]) and some members of this subfamily belong to CE1 in the CAZy database. SF6 contained FAEs from *Chaetomium* sp. CQ31 (ChaeFAE [100]) and *Talaromyces funiculosus* (FaeB [69]) which also belong to CE1 and are closely related to acetyl xylan esterases. SF7 contained exclusively FAEs from *Aspergillus* spp., e.g., *A. niger* (AnFaeA [45]), *Aspergillus flavus* (AfFaeA [101]), and *A. oryzae* (AoFaeA [102]), which are closely related to lipases. SF2–SF4 only contained putative FAEs, which showed sequence similarity to SF1 and tannases (Table 1). Our new phylogenetic analysis classified the putative FAEs into 13 subfamilies (Fig. 2; Additional file 2: Figure S1). In comparison with the previous phylogenetic analysis [51], members of SF1–SF3 and SF5–SF7 remain classified to the same subfamilies. In addition, a FAE from *Fusarium oxysporium* (FoFaeC [103]) has been characterized, which belongs to SF2 and SF7 members that were expanded to cover other fungi than *Aspergillus* spp. (e.g., *Jaapia argillacea*, *Penicillium rubens*, and *Armillaria mellea*). Subfamily SF8 contains FAEs from *Auricularia auricular*-*judae* (EstBC [104]), *Anaeromyces mucronatus* (Fae1A [48]), and *Orpinomyces* sp. (OrpFaeA [105]), while SF12 contains *Pleurotus sapidus* (Est1 [106]) and *Pleurotus eryngii* (PeFaeA [57]) FAEs, for which there were no homologs found in the previous study [51]. The new subfamily SF9 separated from SF4 which previously contained a putative FAE from *A. oryzae* (BAE66413). Three tannases (*A. fumigatus* (XP_748839 [107]), *A. niger* (ABX89592 [108]), and *A. oryzae* (BAA09656 [109])) were positioned in SF11, indicating that the enzymes of this subfamily may actually possess tannase activity or potentially dual-activity and may not be true FAEs. The study also resulted in new subfamilies SF10 and SF13. By contrast, no closely related homologs were found for the FAEs from e.g., *Piromyces equi* (PeEstA [110]), *Piromyces* sp. (FaeA [47]), and *Coprinopsis cinerea* (CcEst1 [111]) and together with the other sequences which are not classified in any group, these are referred to as ungrouped sequences (U1–U10, Fig. 2; Additional file 2: Figure S1). These ungrouped sequences may develop into new subfamilies if homologs for them are discovered.

### Reflection on origin of the different types of FAEs and comparison with ABCD classification

As mentioned before, FAEs evolved from a diverse class of enzymes (e.g., tannases, acetyl xylan esterases, lipases, and choline esterases). Most FAEs have evolved from tannases, as enzymes belonging to the subfamilies SF1-4 and SF9-11 are related to tannases. SF5 and SF6 enzymes show relationship with acetyl xylan esterases, whereas SF7 enzymes are related to lipases. SF12 and SF13 are related to both lipases and choline esterases. Some FAEs are also related to xylanases (GH10 and GH11) and α-l-rhamnosidases (GH78), whereas some show no similarity to any of the above enzymes (Additional file 1: Table S1). Having evolved from different types of enzymes may explain why different FAEs target different hydroxycinnamic acids. While the ABCD classification system provides hints for the specificity of putative FAEs [92], it no longer reflects the evolutionary relationships among different FAEs [51, 52]. In comparison with the ABCD system, SF6 and SF7 contain solely type B and A FAEs, respectively, whereas SF1 contains both type B and C FAEs, and SF5 a mix of type A and D FAEs (Table 1). SF1 and SF5 may be further divided to support ABCD classification when more FAEs from these subfamilies are characterized. In addition, the two new subfamilies SF8 and SF12, which are distantly related to SF7, also contain type A FAEs, whereas FAEs from *Ustilago maydis* (SF13) which are distantly related to SF6 also possess type B activity. Therefore, the ABCD system needs to be revisited and combined with the phylogeny-based classification to provide a well-based system that will help in the identification of different types of FAEs and predict the properties of newly discovered FAEs.

### Prevalence of different types of FAEs in fungal genomes

From the 247 published fungal genomes in early 2015, 155 of them contained putative FAEs (Tables 2a, b; Additional file 2: Figure S2). Approximately 10% of genomes had only one putative FAE and, surprisingly, almost 25 and 5% of the analyzed fungal genomes contained more than 10 and 20 putative FAEs, respectively. The basidiomycetes *Auricularia subglabra* and *Moniliophthora roreri* possessed more than 30 putative FAEs followed by the ascomycetes *A. niger*, *Aspergillus luchuensis* (formerly *A. kawachii*), *Oidiodendron maius*, *Colletotrichum gloeosporioides* with more than 20 putative FAEs. This variation in FAE content could be related to the different abilities of the fungi to degrade feruloylated substrates, which in turn may be related to the presence of such substrates in their natural habitat. However, the multiplicity of putative FAEs identified here could include pseudogenes and the similarity-based method could result in the inclusion of other FAE-related enzymes, e.g., SF11 may also contain tannases. We summarized the prevalence of putative FAEs in industrially and ecologically important fungi in Table 2. Most of these fungi produce more than one type of FAEs. It should be noted that our findings are in agreement with the earlier study reporting that *Trichoderma reesei* (syn. *Hypocrea jecorina*) does not have any putative FAEs in its genome [112], and therefore supplementation of FAEs can significantly increase the saccharification efficiency of an enzyme cocktail from *T. reesei* [30, 31, 67]. However, two other species of this genus, *T. atroviride* and *T. virens*, contain three putative FAEs in their genomes.

**Table 2:**
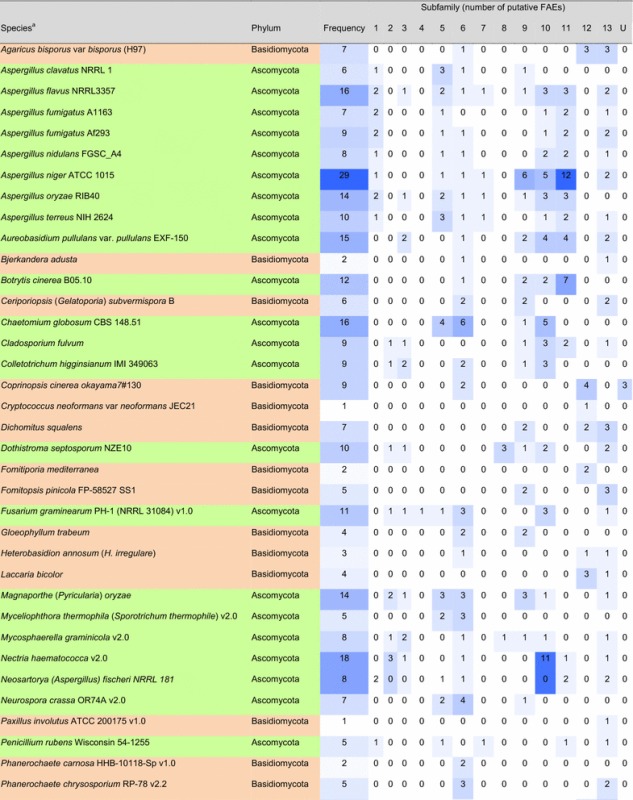

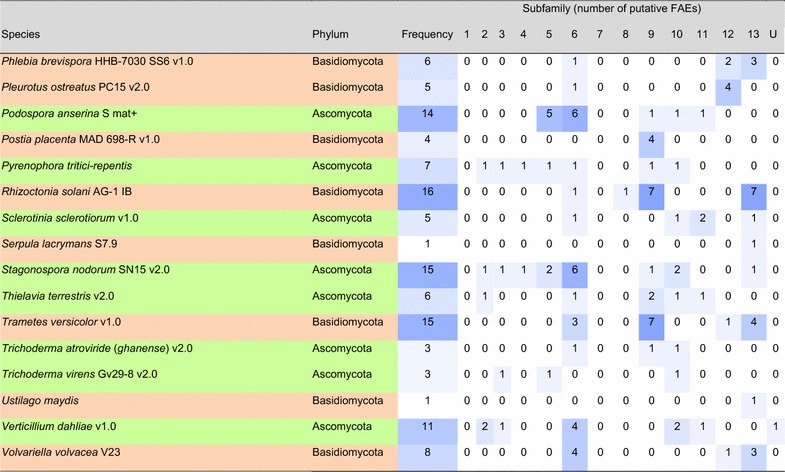
Prevalence of the families of FAEs in industrially and ecologically important fungal genomes

Intensity of blue color indicates the frequency of (putative) FAEs in the subfamily

*U* ungrouped sequences

^a^Current name is shown in parenthesis

## Industrial applications of FAEs

**Fig. 3:**
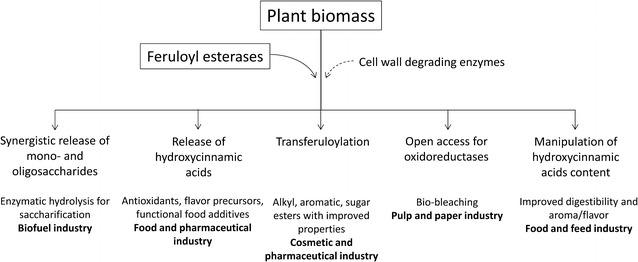
Schematic overview of industrial applications of FAEs (modified from [51])

With the ability to remove hydroxycinnamic acids from plant cell walls, FAEs have considerable roles in biotechnological processes for various industrial applications. Earlier Fazary and Ju [113] excellently reviewed the early industrial use of FAEs through patents. To date the patents on FAE applications and discovery are almost doubled compared to 2008. In this section, we update the patents on FAEs presented in European Parliament documents (EP) and World Intellectual Property Organization-Patent Cooperation Treaty (WIPOPCT) databases (Additional file 1: Table S3), and highlight the applications in five major fields: (1) biomass processing, (2) ferulic acid and related fine chemicals production, (3) pulp and paper, (4) feed and (5) seasonings and alcoholic beverages (Fig. 3).

### Applications in biomass processing

FAEs are considered to be essential accessory enzymes to complete hydrolysis of lignocellulosic biomass for bioethanol and other biorefineries. To date more than 150 patents have been filed on applications of FAEs towards biomass processing (Additional file 1: Table S3, both discovery and saccharification). Activity of FAEs on plant biomass in combination with other hydrolases and oxidases not only significantly increases the breakdown of plant materials and enhances the availability of fermentable carbohydrates, but it also releases phenolic compounds and toxic esters which inhibit the fermentation process of pretreated lignocellulosic materials (e.g., [114]). For this reason, fusions of FAEs and other enzymes/proteins have also been created aiming to increase the catalytic efficiency and/or substrate affinity [115–117]. Different strategies have been applied to create FAE mutants which can tolerate the high temperatures in bioprocesses [118–120]. Furthermore, transgenic plants have been manipulated specifically for biofuel production to reduce recalcitrance of cell walls prior to saccharification, which also enhance the digestibility and biomass conversion for livestock (e.g., [32–35, 121]). Besides, FAEs are not only used for complete hydrolysis of lignocellulosic materials, but they can also be applied for manipulating the structure of oligosaccharides e.g., in production of *xylo*-oligosaccharides [122] which are industrially important functional food additives with prebiotic properties [123].

### Applications in production of ferulic acid and related fine chemicals

Ferulic acid and other hydroxycinnamic acids are phenolic phytochemicals which are widely used in food and cosmetic industries because of their unique and potent properties as, e.g.,

1. Antioxidant—they are able to neutralize free radicals, e.g., reactive oxygen species which are implicated to cause DNA damage, cancer, and accelerated cell aging [124–126].
2. Sun protection factor—they are able to absorb UV radiation by the presence of conjugated double bonds, e.g., in an aromatic structure [124, 127].
3. Depigmenting agent—they are tyrosinase inhibitors because their chemical structures resemble those of tyrosine and are suggested to prevent the formation of melanin by competitive inhibition with tyrosine [128, 129].
4. Precursor for synthesis of flavor compounds, such as vanillin and guaiacol (e.g., 4-vinyl guaiacol)—intermediates of ferulic acid degradation pathway. These intermediates are of great interest in the food and fragrance industry [130, 131].

Ferulic acid and other hydroxycinnamic acids can be used as a carrier of vitamin C and E, which double their skin photoprotection with stronger lipophilicity allowing better penetration into the stratum corneum [132]. Furthermore, they show pharmaceutical and health beneficial functions, e.g., antimicrobial, anti-inflammatory, anti-diabetic, anti-thrombosis, anti-cancer, and cholesterol-lowering agents [11, 12, 133]. Although commercially ferulic acid is mainly produced from rice oil (as γ-oryzanol), modern processes are focusing on production of ferulic acid by FAEs in combination with other hydrolases in a biorefinery process (e.g., [121, 122]).

**Fig. 4:**
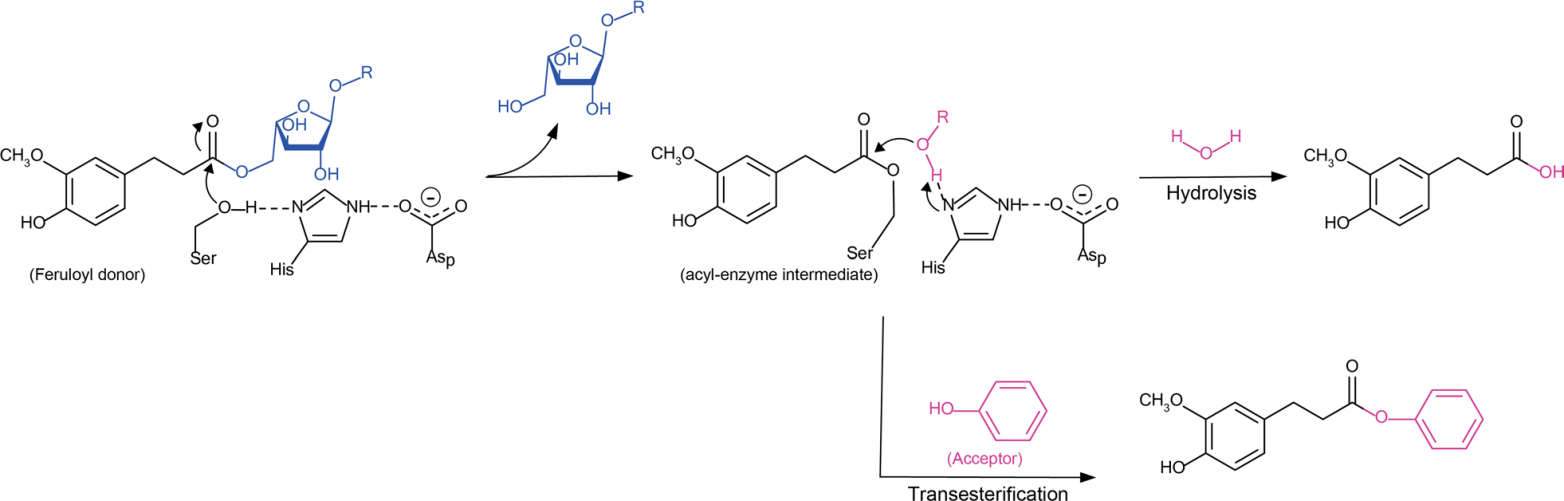
Transferuloylation reaction (modified from [64])

Apart from hydrolysis, FAEs can be used for synthesis of ester-linked hydroxycinnamic acids through a transesterification reaction by exchanging the organic group of an ester (donor) with the organic group of an alcohol (acceptor) (Fig. 4), to obtain products with altered chemical and biological properties. The first report on transesterification activity of FAE was investigated on FAE from *Sporotrichum thermophile* (StFaeC) using arabinose and arabinobiose as acceptors [134]. Containing both hydrophobic ferulic acid and hydrophilic oligosaccharide moieties, feruloylated arabinose and oligosaccharides possess the physiological functions of both. This includes antioxidant activity, probiotic effects, and inhibition against glycation which are of interest by a wide range of applications in food, pharmaceutical, and cosmetic industries [135]. The advantage of using transesterification over hydrolases or transferases is the flexibility of their acceptor molecules, which can vary from different carbohydrates [66, 136, 137], aliphatic and aromatic alcohols [138, 139], and glycerol [140, 141] to propolis [142]. In the latter case, FAEs can also be used for impoverishing the allergenicity of propolis by specifically removing esters of caffeic acid under hydrolytic conditions [143].

### Applications in pulp and paper industry

To produce high-quality paper, whiteness is an important characteristic of wood pulp. Discoloration of the pulp is caused by lignin remaining in the pulp and bleaching is the key step to whiten the pulp by removing the residual lignin. This process uses hazardous and expensive chemicals; mainly chlorine dioxide and hydrogen peroxide or ozone in elemental chlorine-free (ECF) and in totally chlorine-free (TCF) chemical processes, respectively. In the environmentally friendly biobleaching process, FAEs can be used in combination with xylanases and lignin-oxidizing enzymes [144–146] particularly in a bi-sequential process reported by Record et al. [144], which the delignification rates were comparable to the results obtained with hazardous chemicals. The enzymatic process also resulted in lower energy consumption and a significant reduction of the chemical oxygen demand (COD) value of the pulping waste water [147].

### Applications in feed industry

Fiber digestibility is an essential criterion for animal feed. Suffering from improper digestion can hamper animal growth and cause immunological stress which results in reduction of the feed conversion ratio in livestock, and hence restricts profitability of farmers. Ferulic and hydroxycinnamic acids themselves can promote health in animals [148, 149]; however, feruloylation in plant cell walls particularly in a high forage diet is among the major inhibitory factors for the ruminant digestive system. Addition of FAEs or enzyme cocktails containing FAEs can improve the access of main chain degrading enzymes resulting in improved fiber digestion and bioavailability of phytonutrients, accelerating animal growth (e.g., [150, 151]), as well as reducing immunological stress [152].

### Applications in seasonings and alcoholic beverage industry

Surprisingly, FAEs have been used for both removing off-flavors/odors as well as enhancing the aroma in several seasonings and alcoholic beverages. Flavor and odor are the crucial ingredients for success in the premium fermented seasonings and alcoholic beverage industries in particular Japanese rice wine and cooking liquor—sake and mirin. The major flavor component from these products is ferulic acid as well as its derivatives including 4-vinyl guaiacol, vanillic acid, and vanillin. FAEs can be applied in the saccharification process as a FAE-producing koji (rice-fungal culture starter) or an additive together with xylanases and cellulases to increase the release of ferulic acids from the cell wall of rice and other cereal grains, which then can be converted to the aromatic derivatives during the fermentation and aging process [54, 153–155].

### Other applications

Apart from the above-mentioned applications (Additional file 1: Table S3), FAEs can also be used (1) in a form of live FAE-producing Lactobacilli supplement which can reduce triglyceride concentrations, hepatic inflammation and insulin resistance in medical applications [133, 156]; (2) in the milling process for starch production, where FAE is used during the wet milling together with e.g., cellulase and proteases providing an increase in production yield [157]; and (3) in detergent applications, where FAE-containing multi-enzyme system is used to improve the performance of liquid laundry detergents particularly at low temperature (e.g., [158]).

## Conclusion

In this review, we provide insight into biodiversity, biochemical properties, production, and discovery of FAEs, a highly diverse group of plant cell wall degrading enzymes. Although FAEs generally play a role in catalyzing the release of ferulic acid and other hydroxycinnamic acids from plant cell wall polysaccharides, they possess diverse specificities towards different feruloylated poly- and oligosaccharides and monomeric hydroxycinnamates. FAEs have evolved from different types of enzymes (e.g., tannases, acetyl xylan esterases, and lipases), which is reflected by their amino acid sequences. Classification based on phylogenetic analysis divided FAEs into distinct groups and also resulted in discovery of novel putative FAEs. These new FAE candidates may possess different substrate specificities and/or biochemical properties which may be useful in different applications. It is clear that more biochemical characterization of FAEs is needed for better understanding of substrate specificity and mode of action of FAEs from different subfamilies. The range of industrial applications of FAEs has been broadened over the past years with emphasis on the conversion of agro-industrial waste materials into valuable products and the synthesis of novel ester-linked hydroxycinnamic products in particular for health and cosmetic applications. The industrial uses of FAEs are still limited to only a few enzymes. Here, we provided the phylogenetic-based classification and putative FAEs resulting from genome mining as a guideline for exploration of FAEs towards the specific applications.

## Additional files

**Additional file 1: Table S1.** Substrate specificity, biochemical properties, induction conditions, classifications based on the amino acid sequences, **Table S2.** The prevalence of the FAE families in all fungal genomes used in this review article, **Table S3.** Patents and the inventions related to FAE applications, **Table S4.** Sequences of characterized and putative FAEs used in this study.

**Additional file 2: Figure S1.** Phylogenetic tree of the (putative) fungal FAEs. FAEs from previously reported phylogenetic analysis [51] were marked with magenta open triangles for SF1, magenta open rhombuses for SF2-4, magenta filled triangles for SF5, magenta filled rhombuses for SF6, light blue filled squares for SF7, and magenta filled circles for ungrouped ones. AtFAE2 and AtFAE3 are marked with brown filled circles, acetyl xylan esterases are marked with blue filled circles, lipases are marked with yellow filled squares, tannases are marked with purple filled circles, glucuronoyl esterases (as an outgroup) were marked with green filled circles. The same symbols are used in Fig. 2.

## Abbreviations

CAZy: carbohydrate-active enzymes database
FAE: feruloyl esterase
SF: subfamily
