# Supplementary figures and images for: Diversity of fungal feruloyl esterases: updated phylogenetic classification, properties, and industrial applications
Source: Biotechnol Biofuels. 2016 Oct 28;9:231. doi: 10.1186/s13068-016-0651-6 (PMC5084320; doi:10.1186/s13068-016-0651-6)

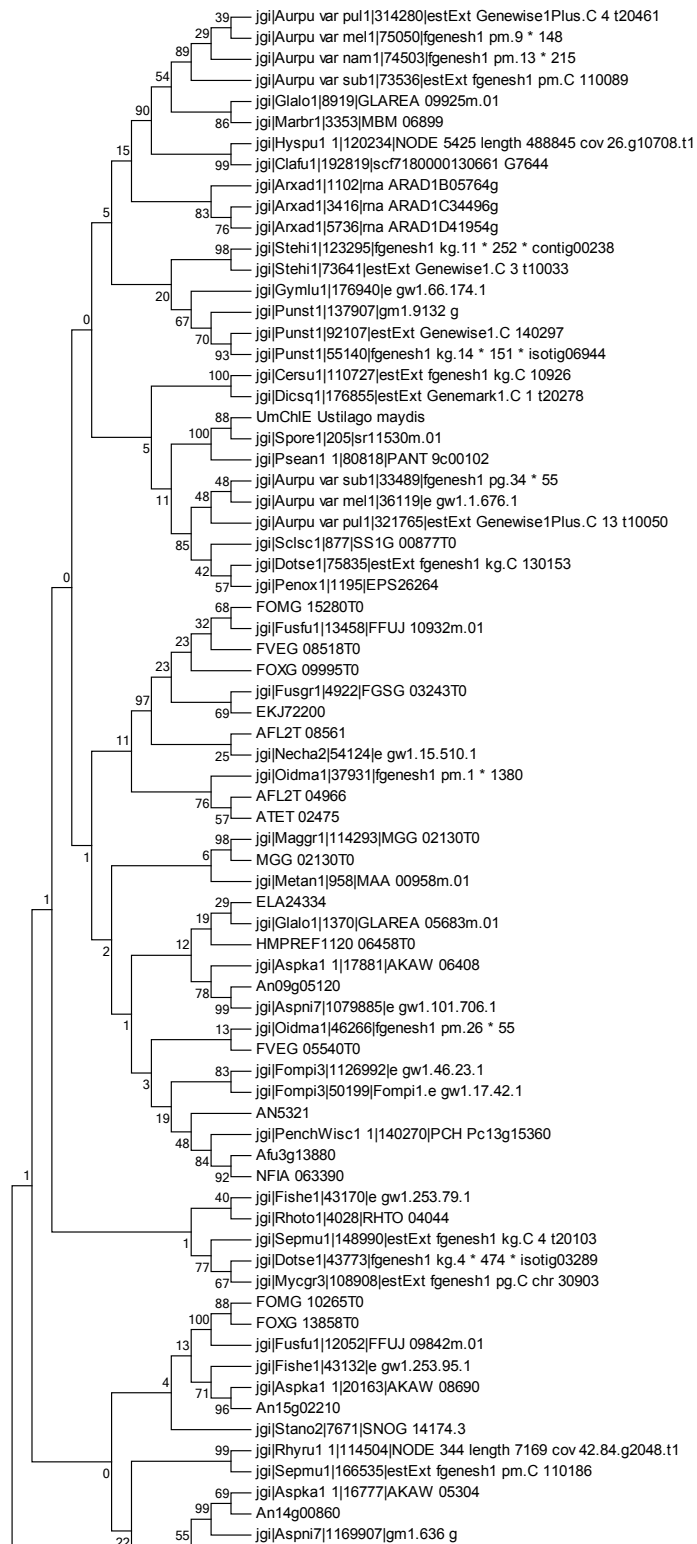

SF13

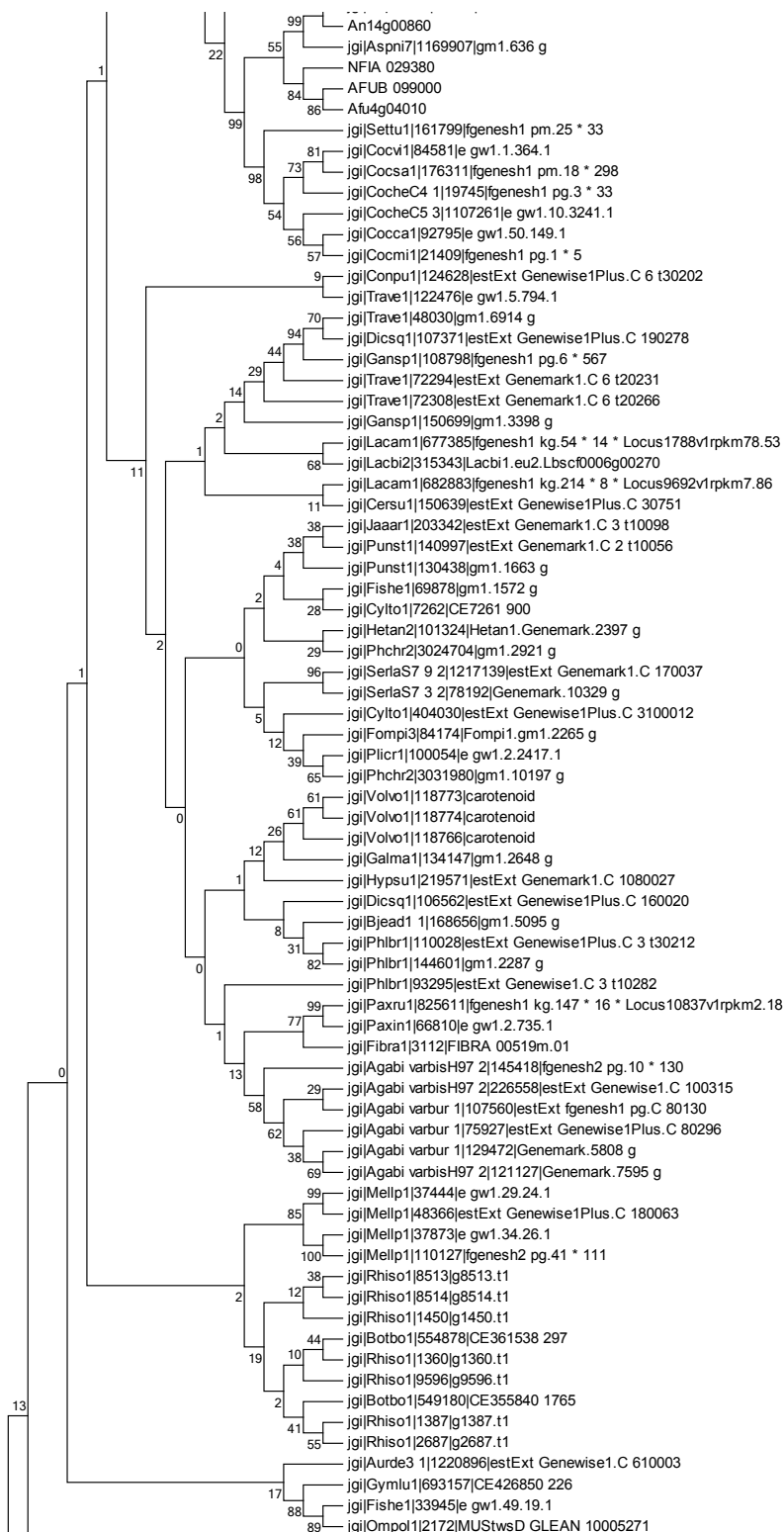

SF13

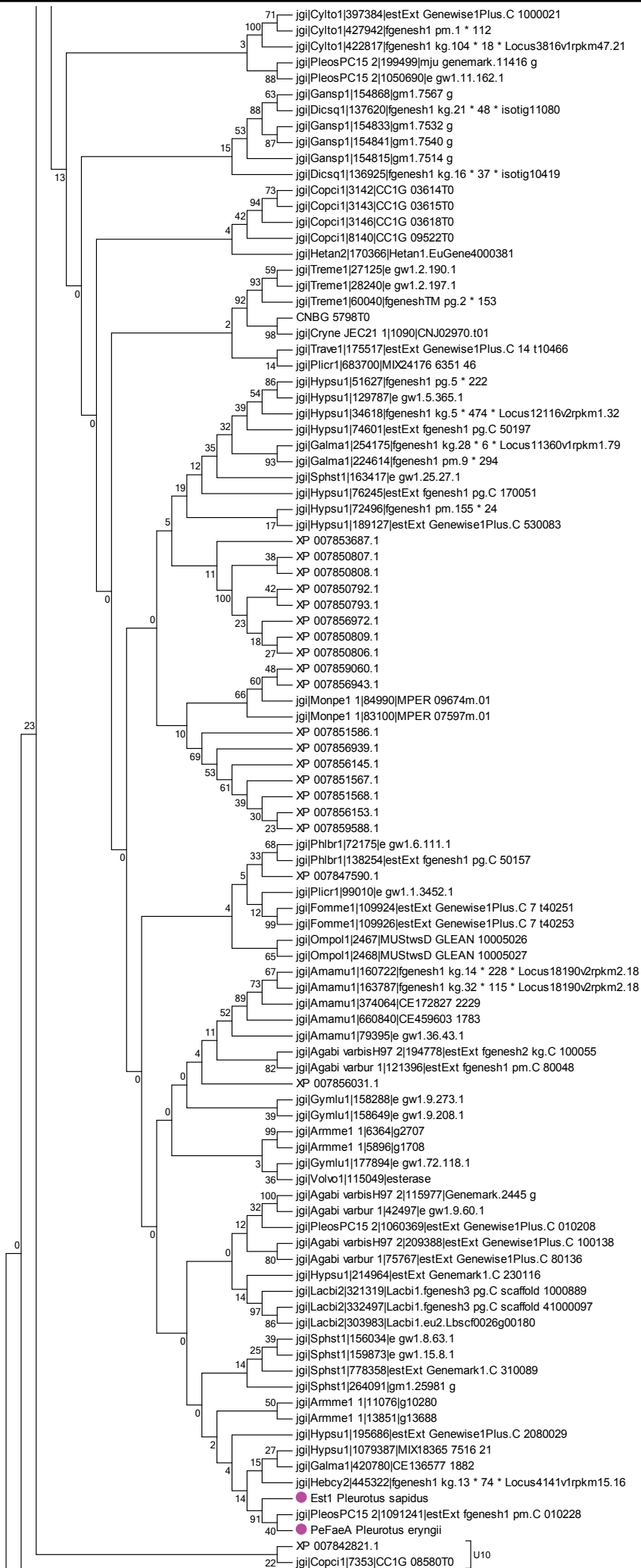

SF12

(SF12, U10)

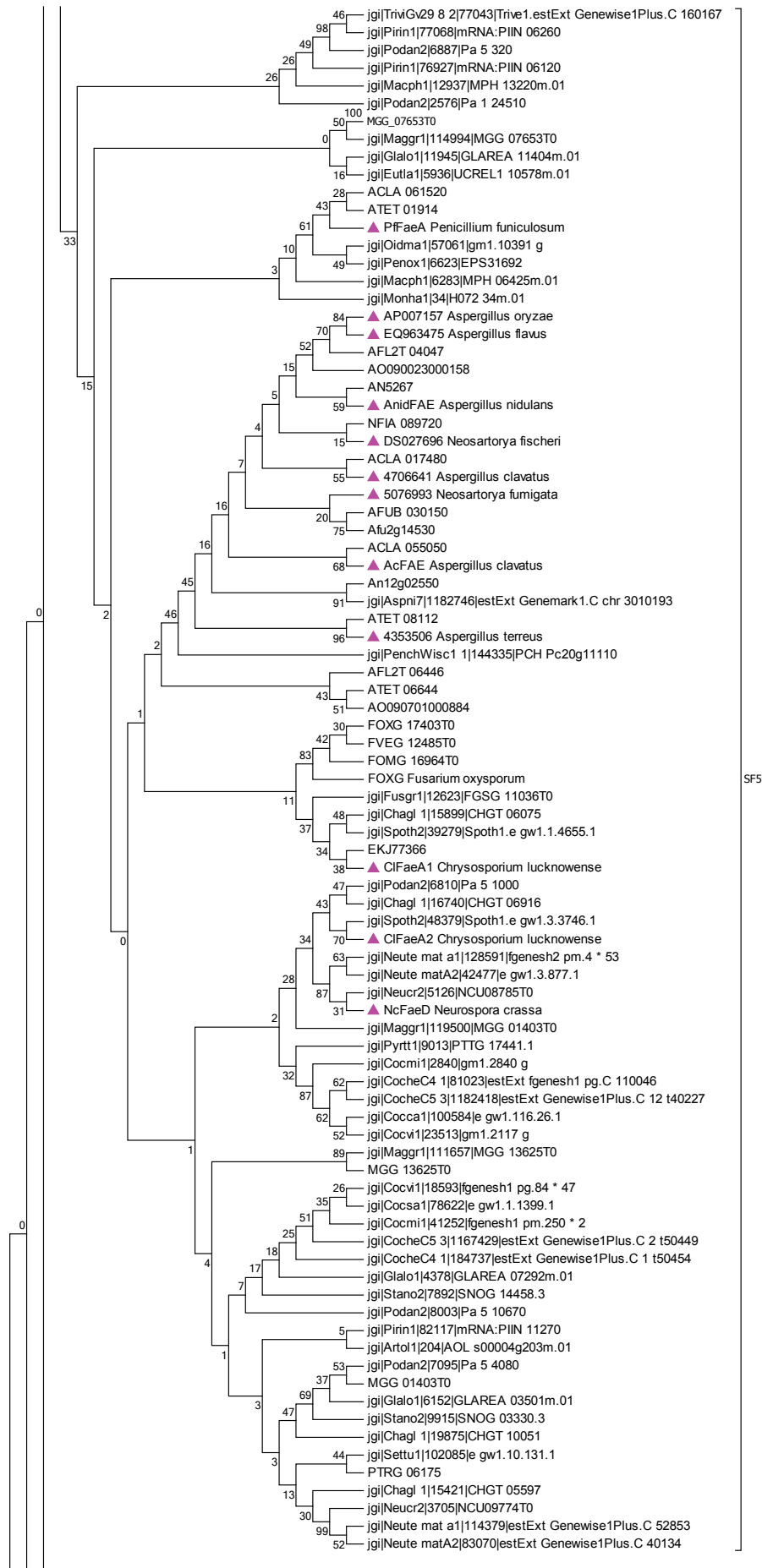

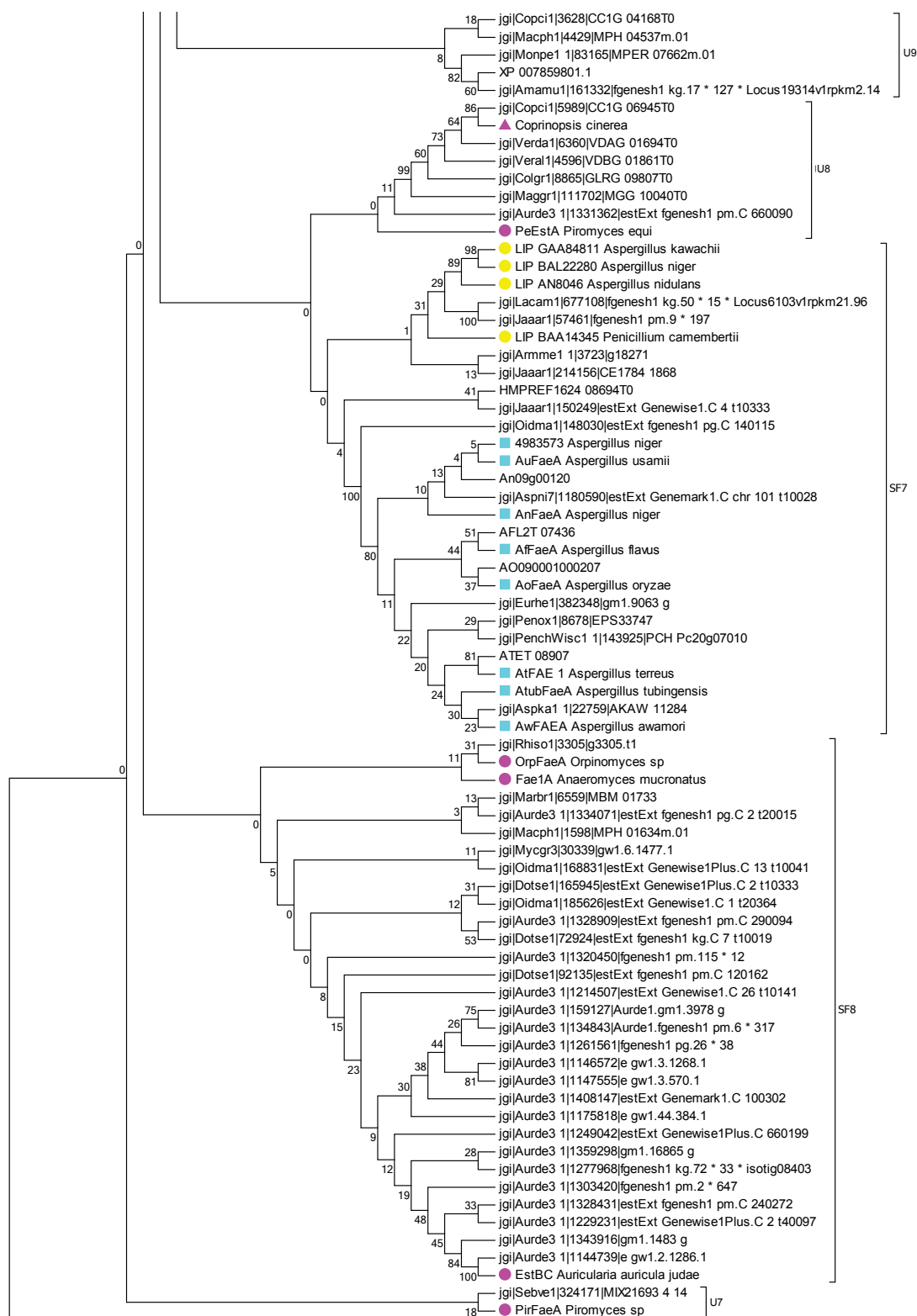

(SF7, SF8, U7-U9)

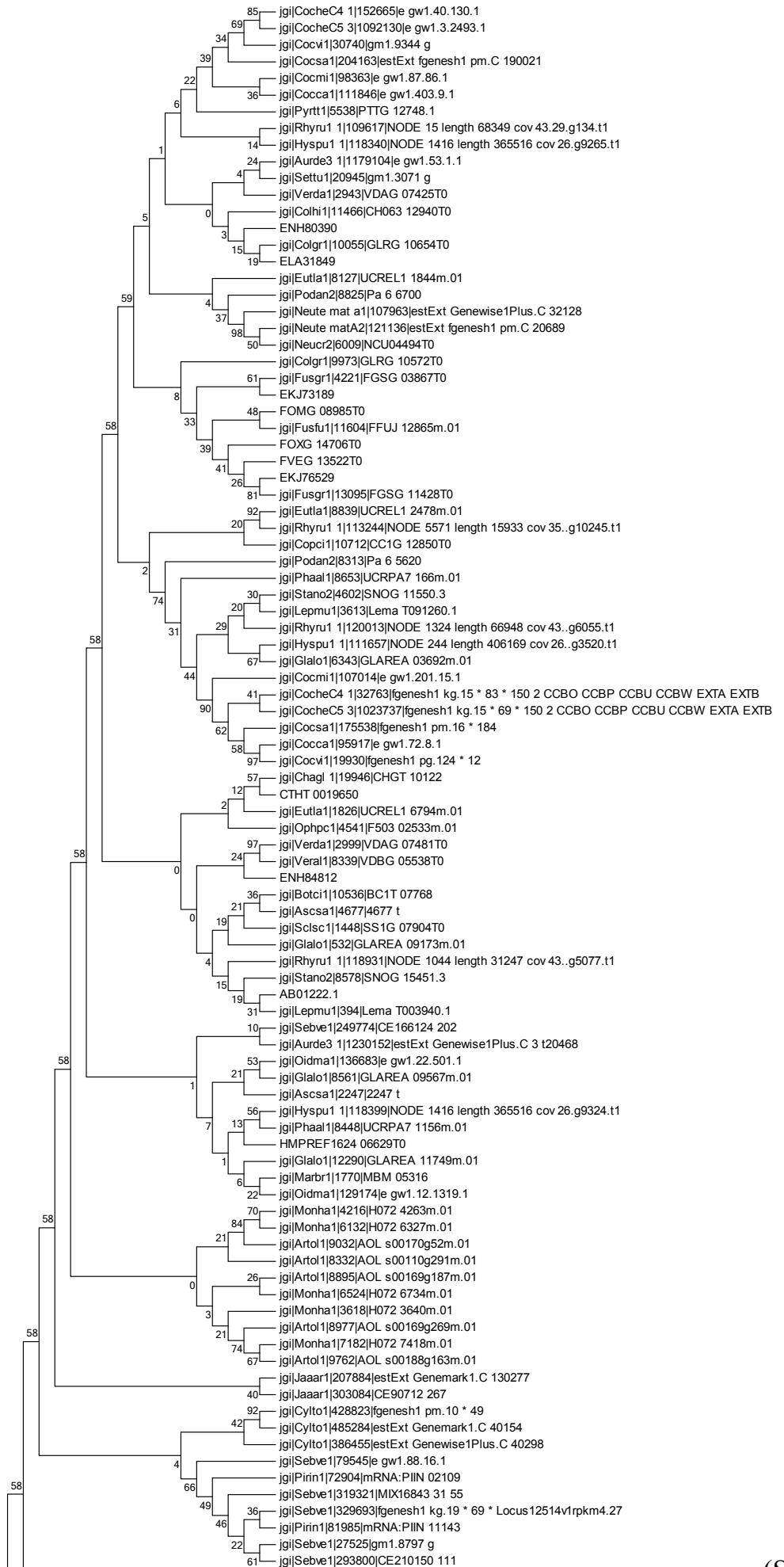

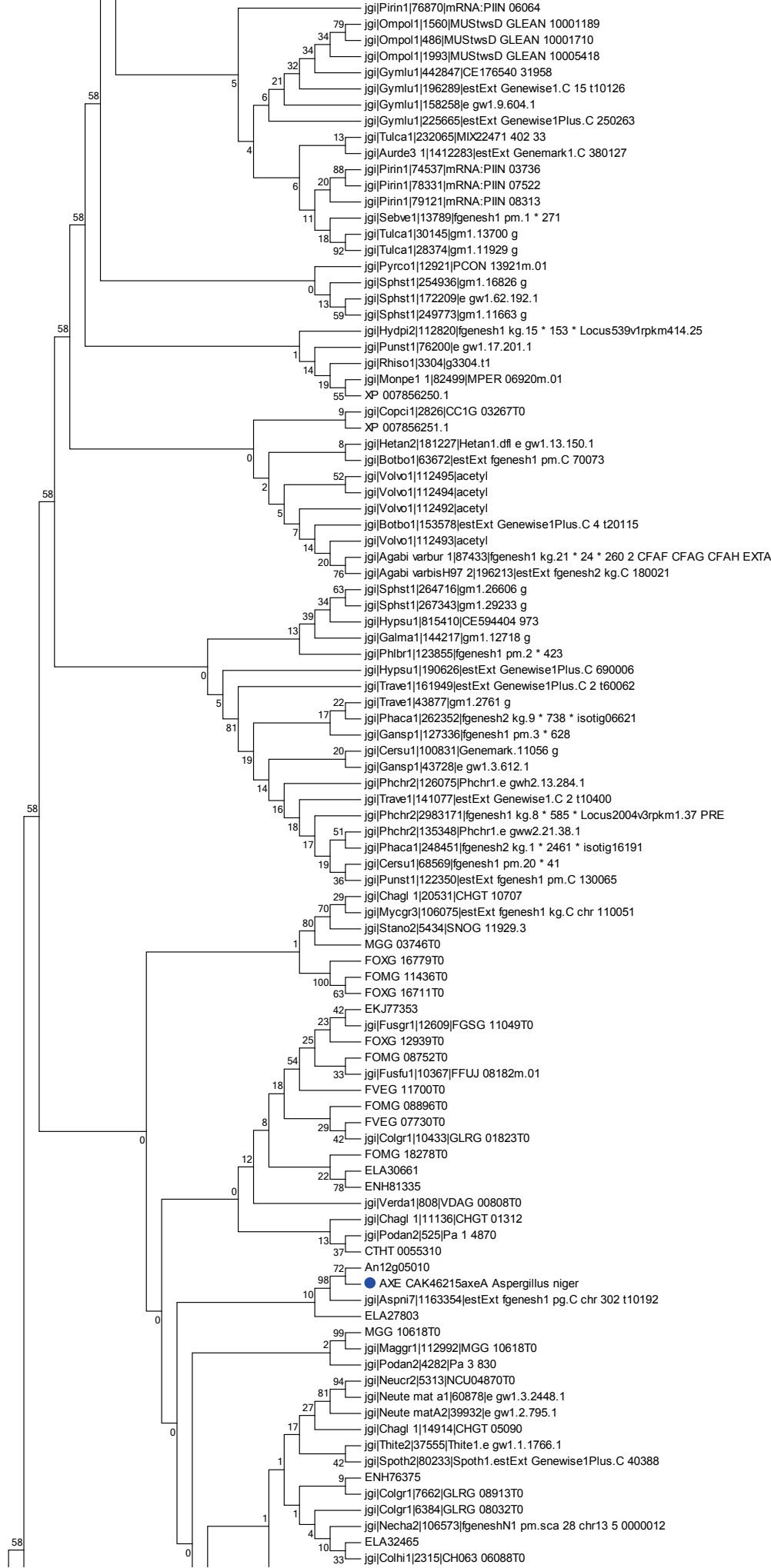

SF6

(SF6)

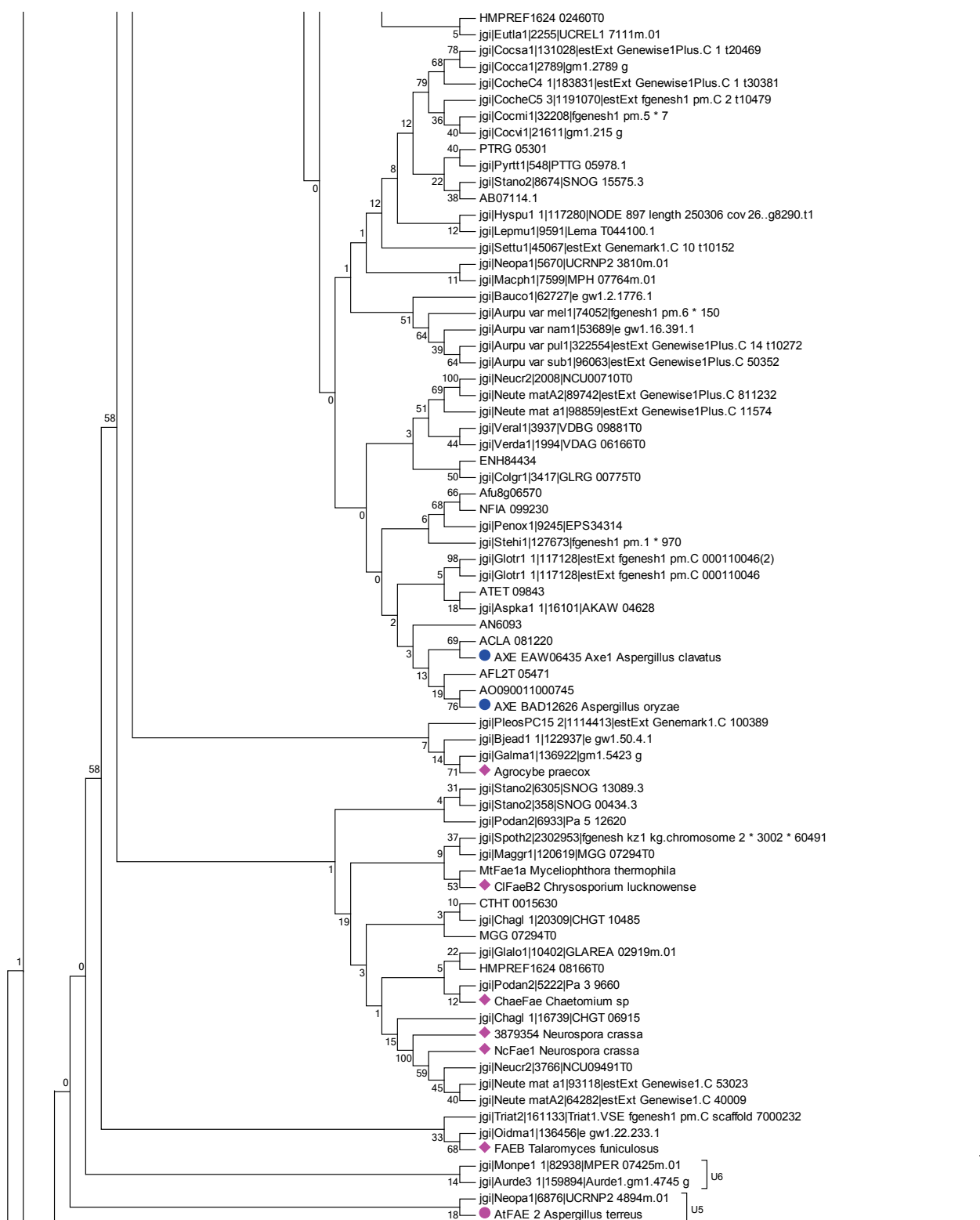

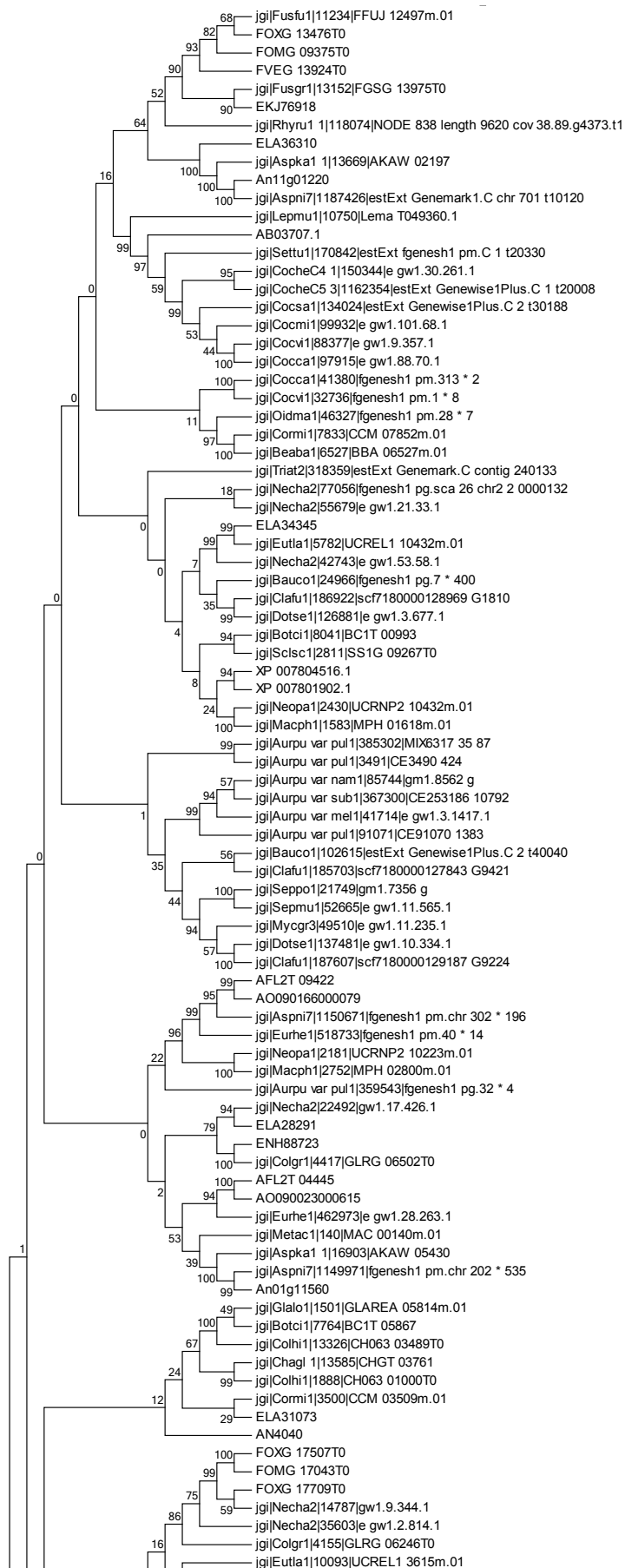

SF10

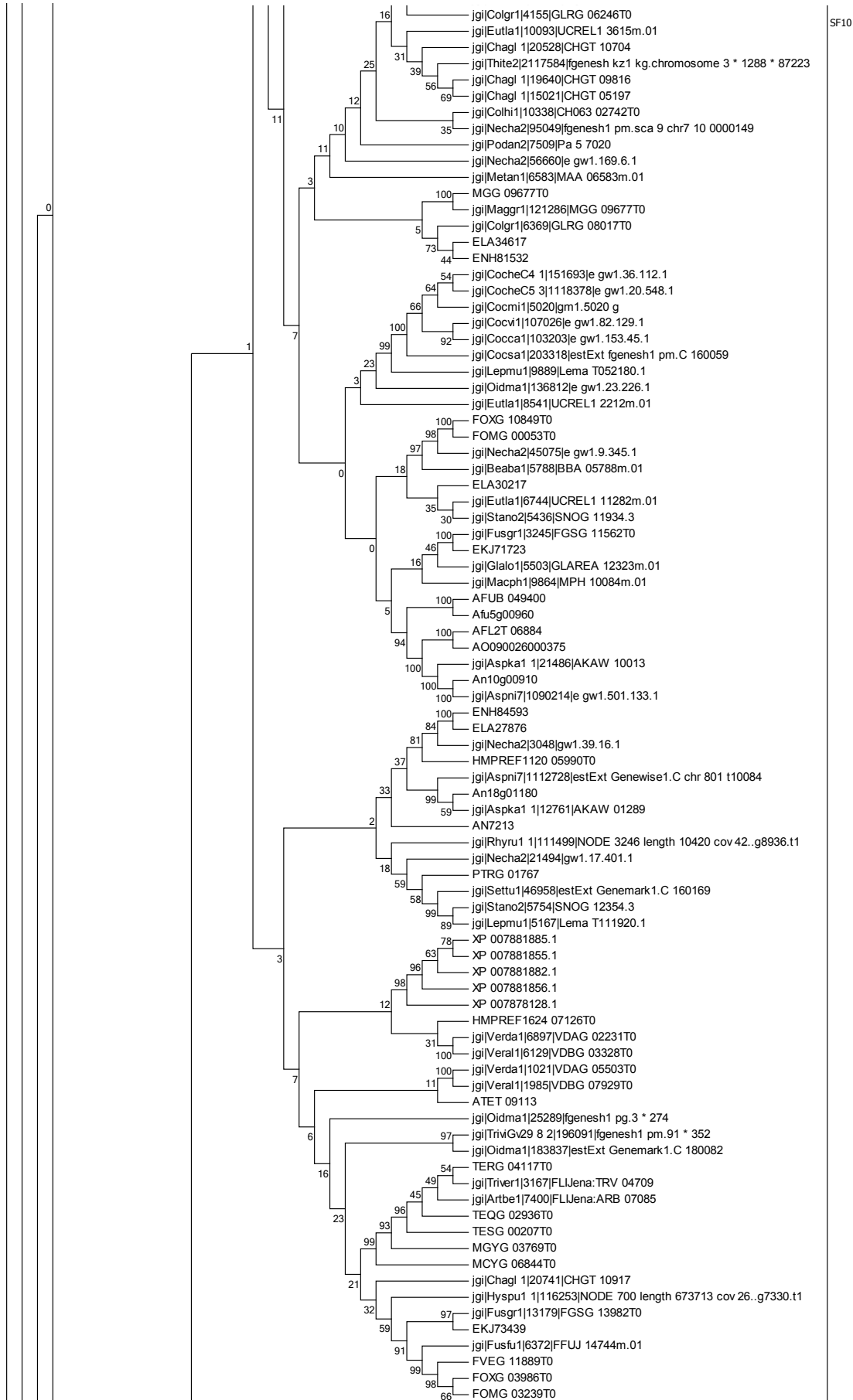

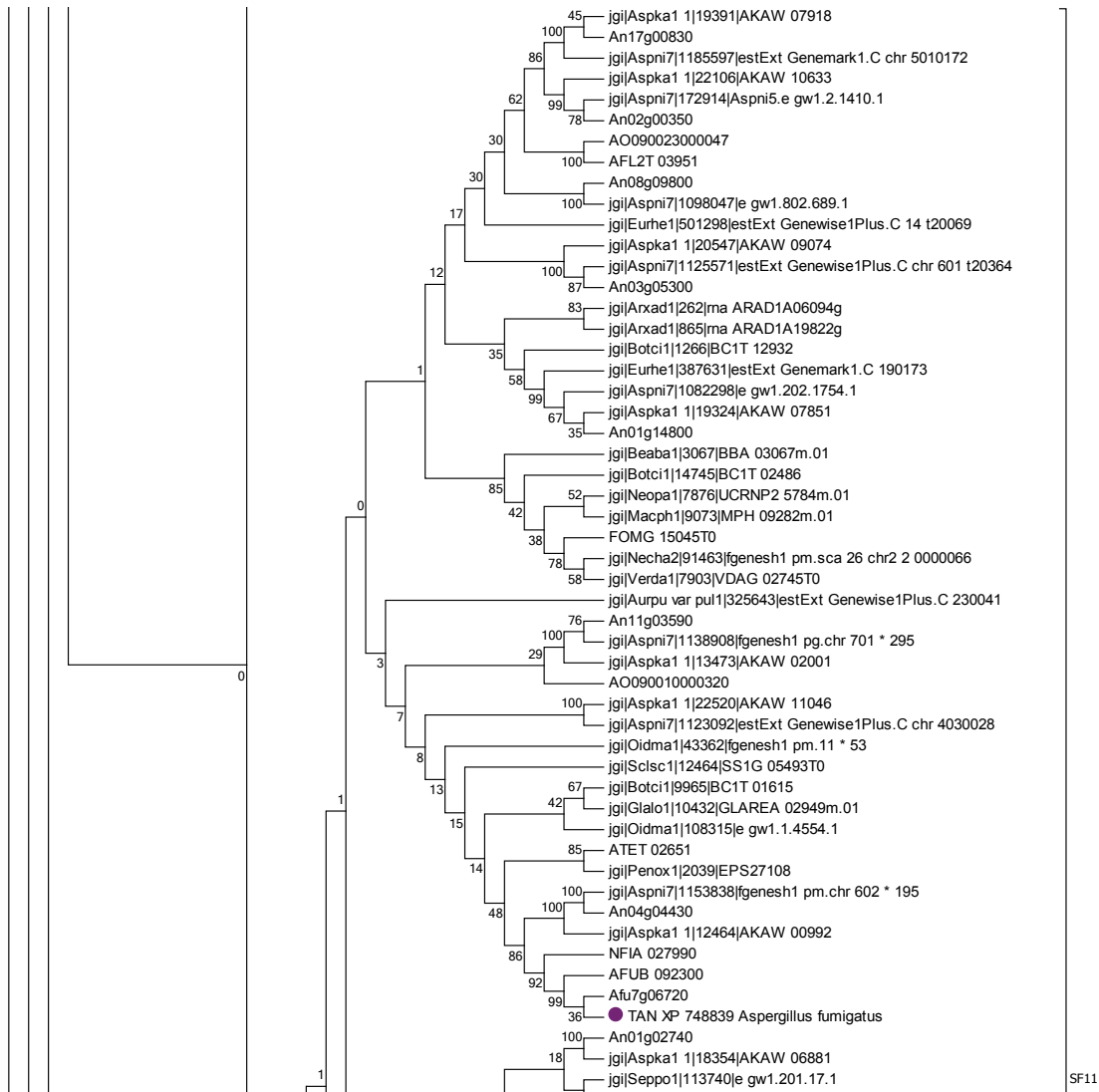

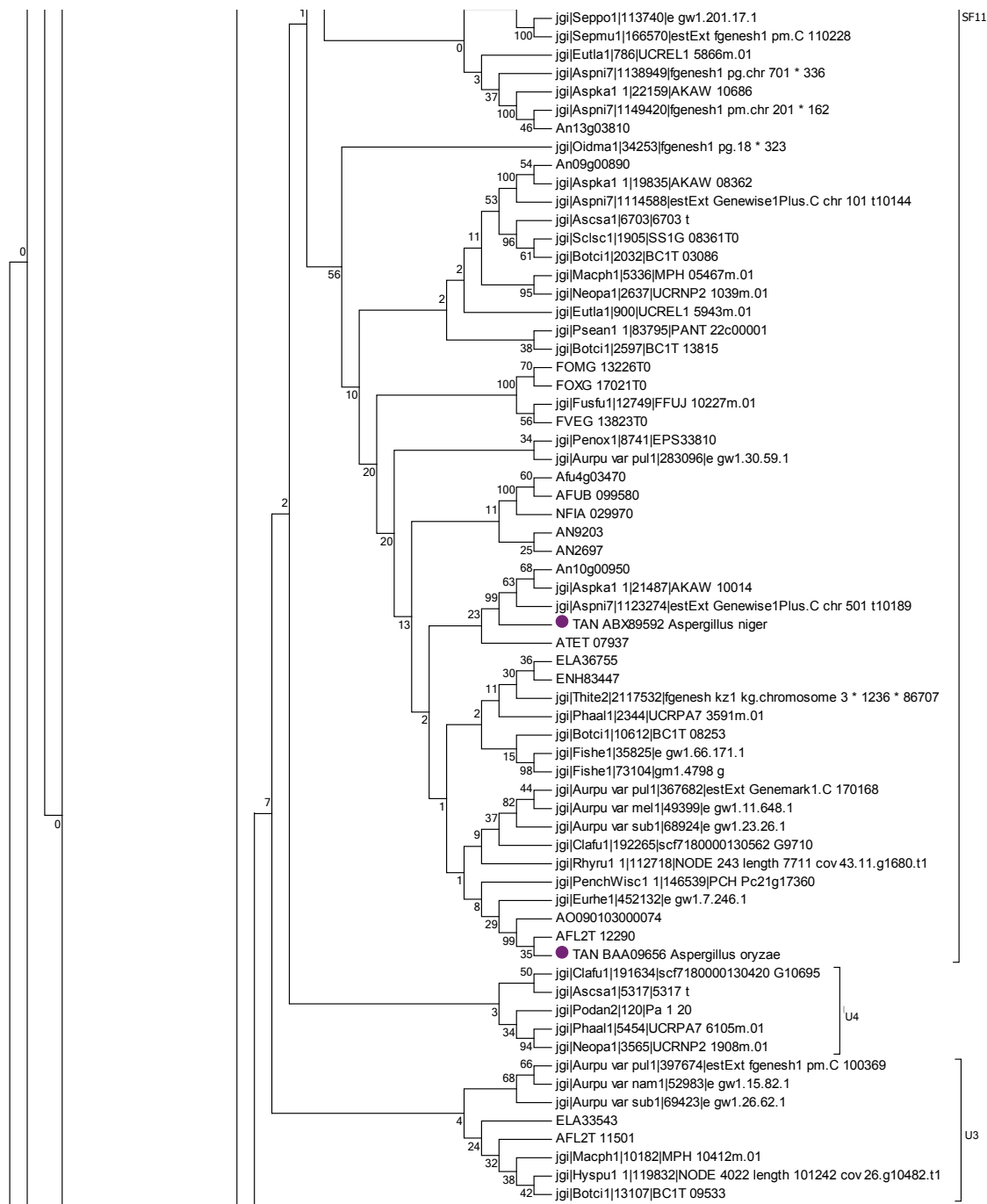

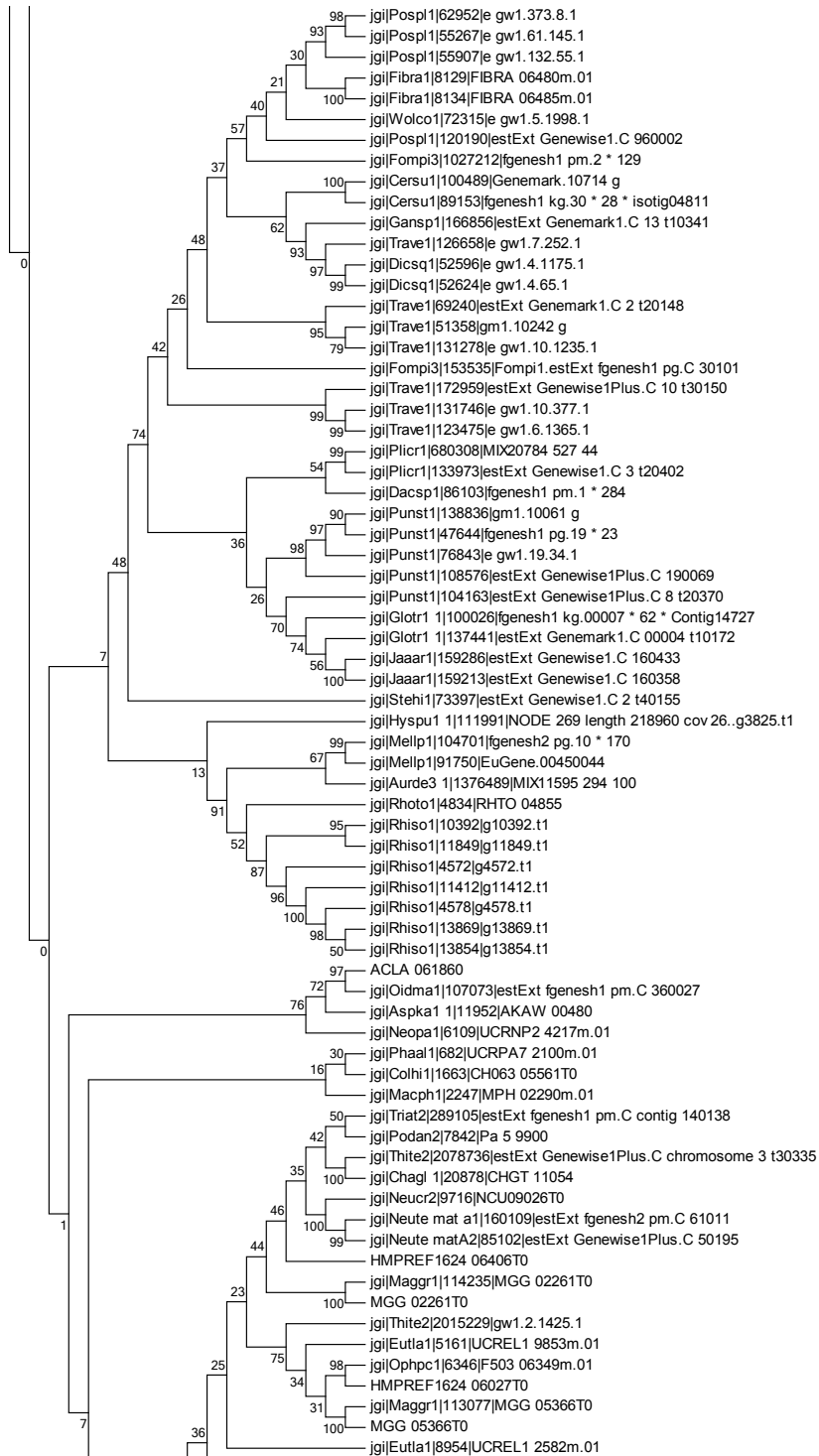

SF9

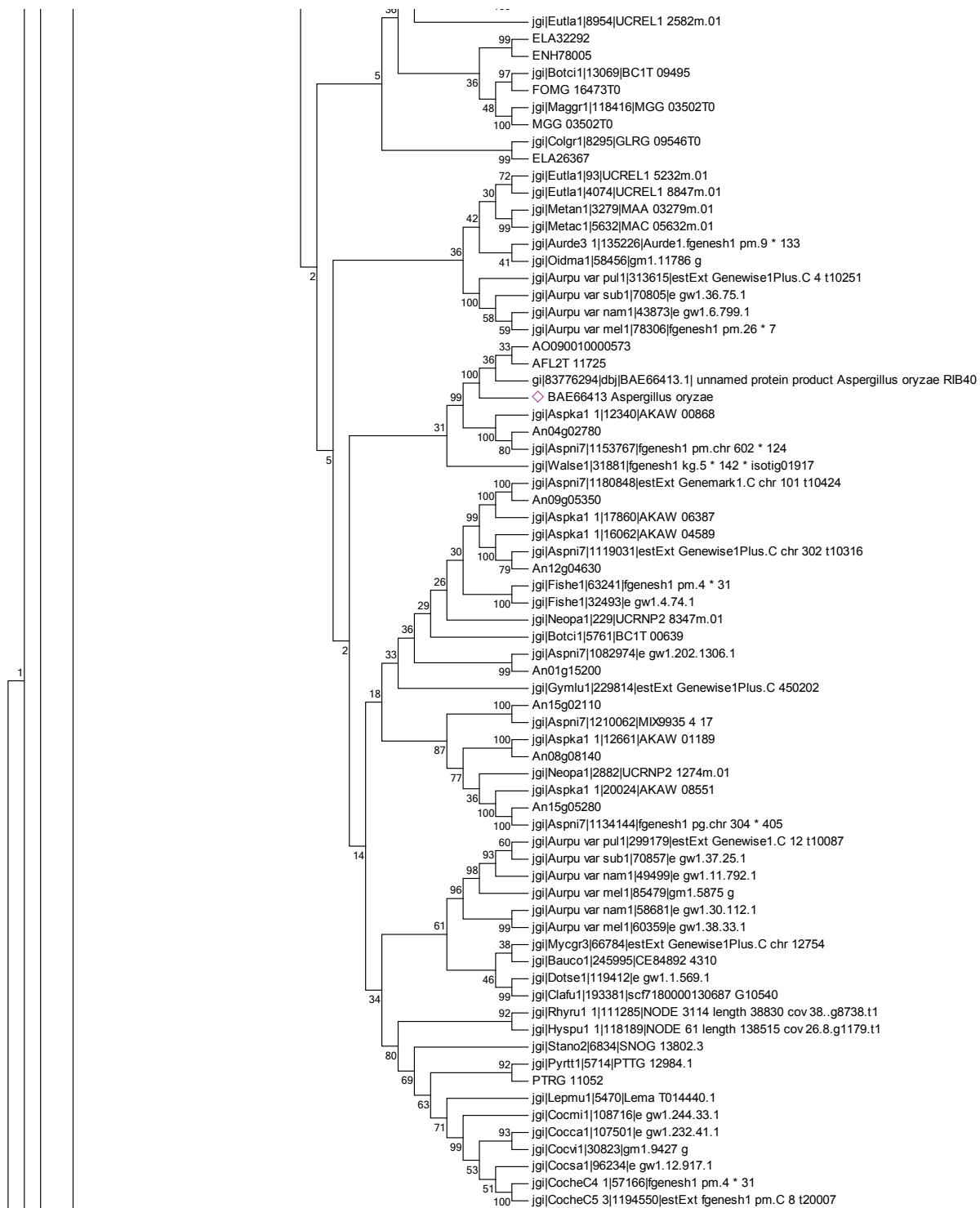

SF9

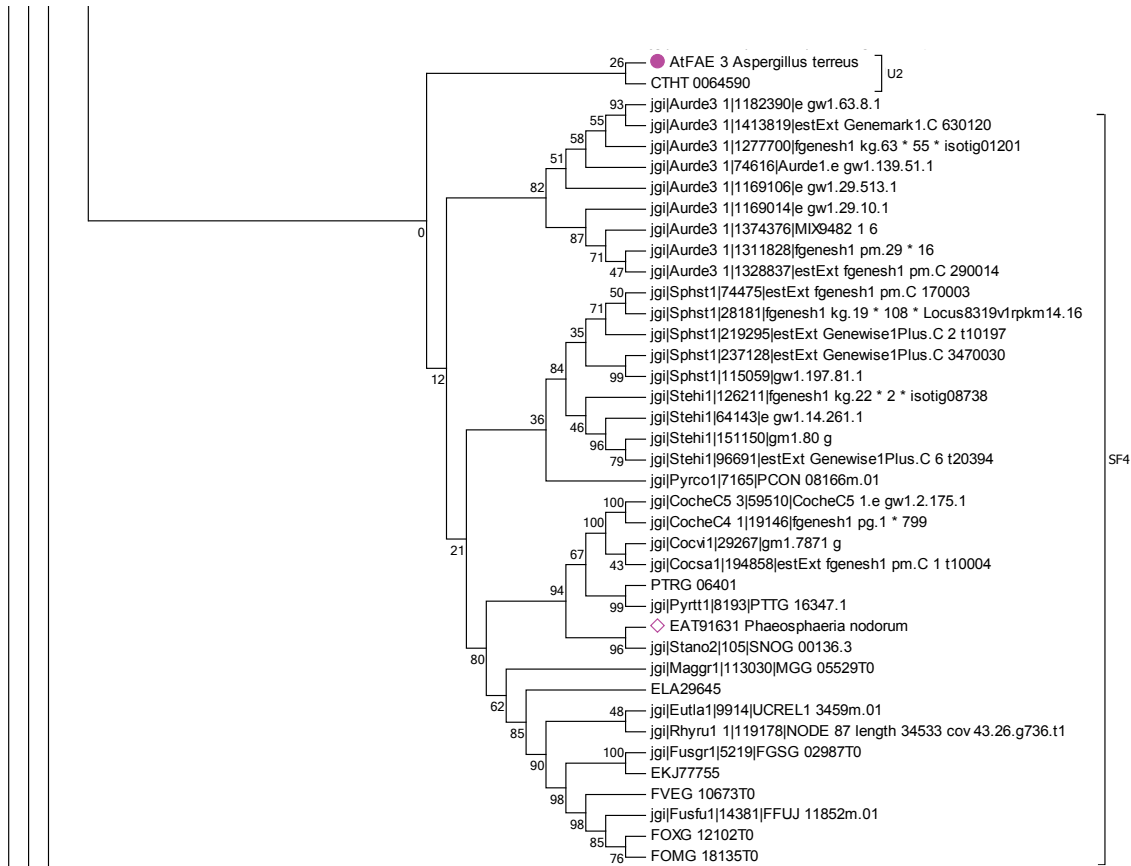

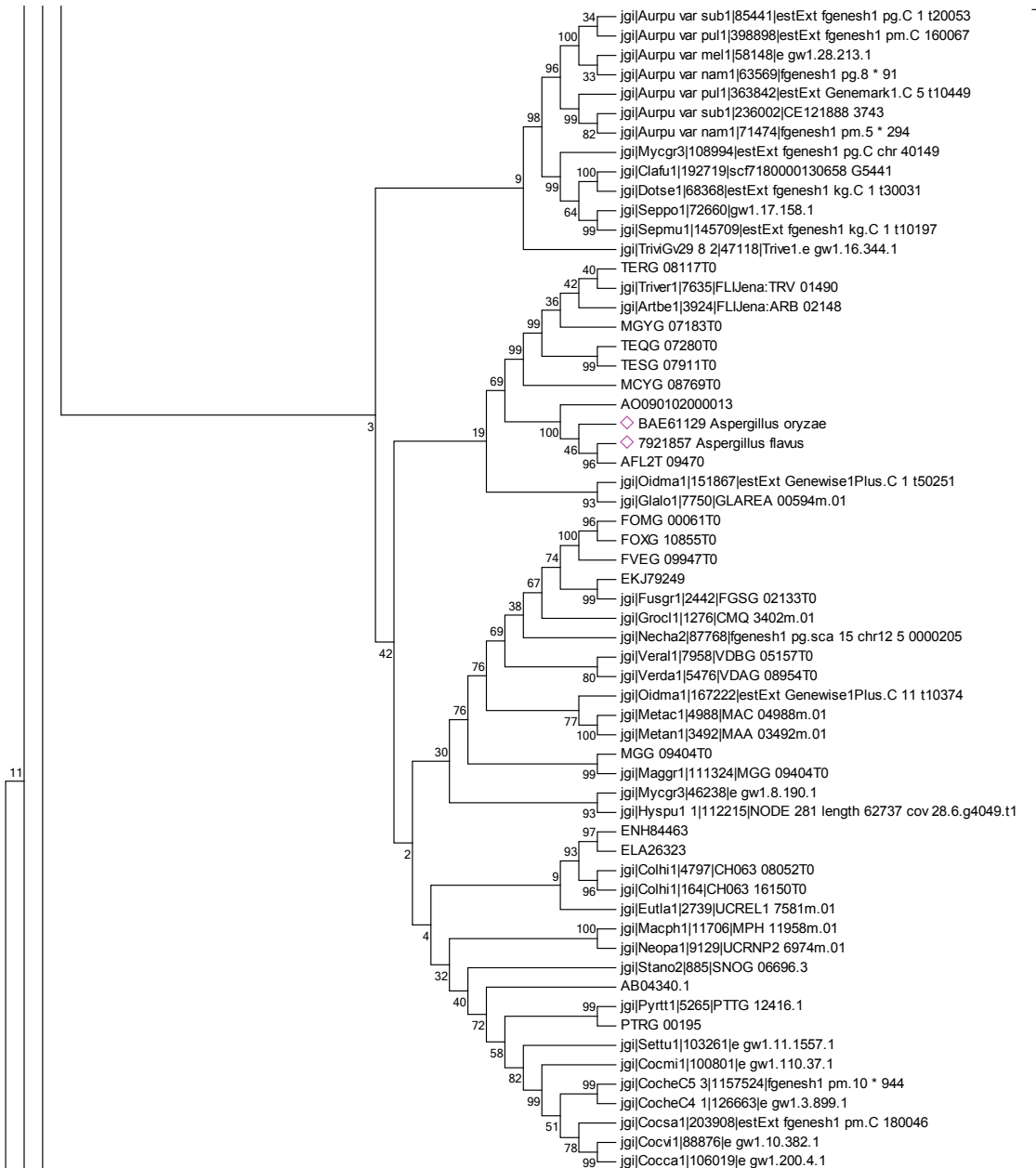

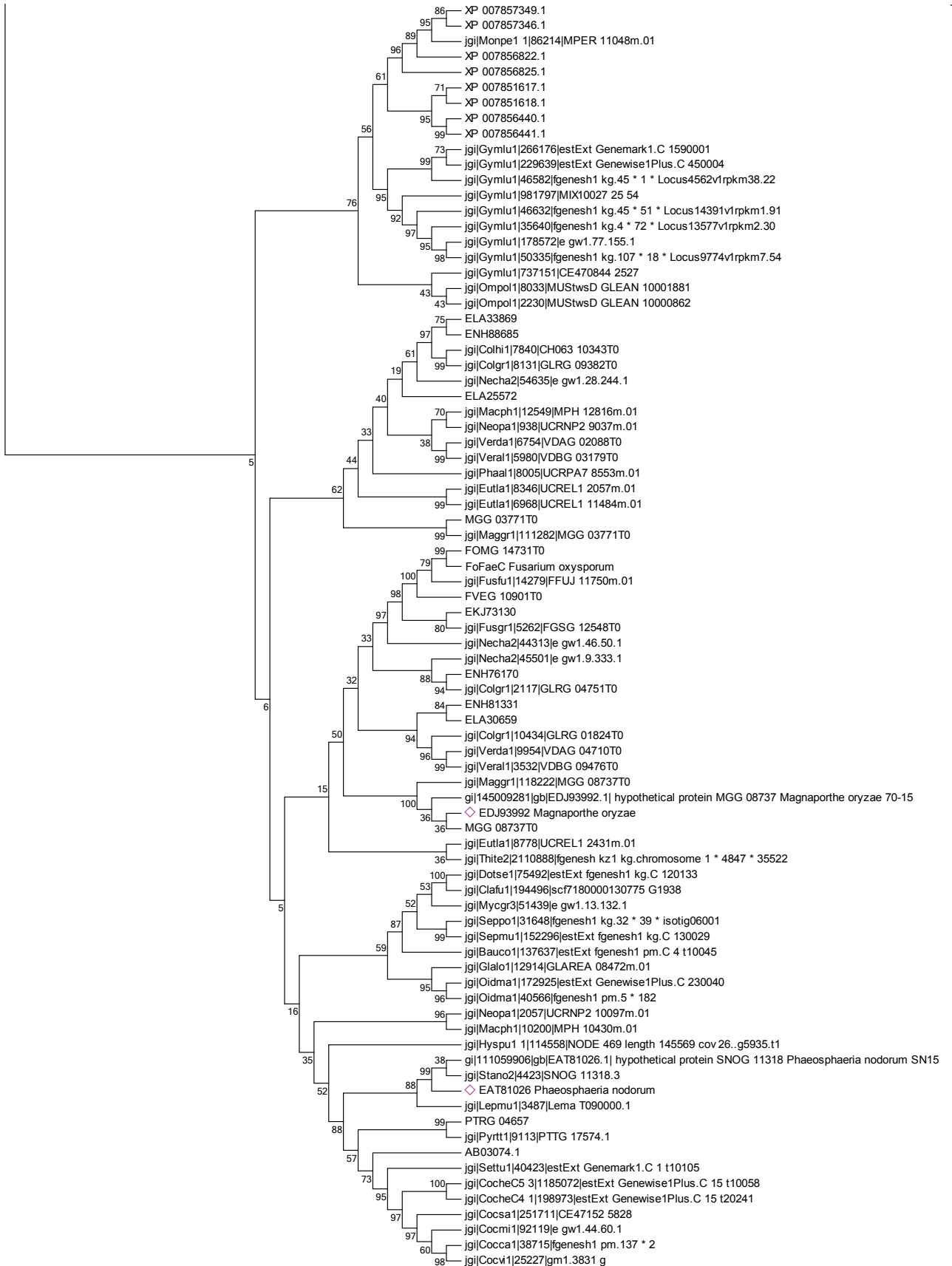

SF2

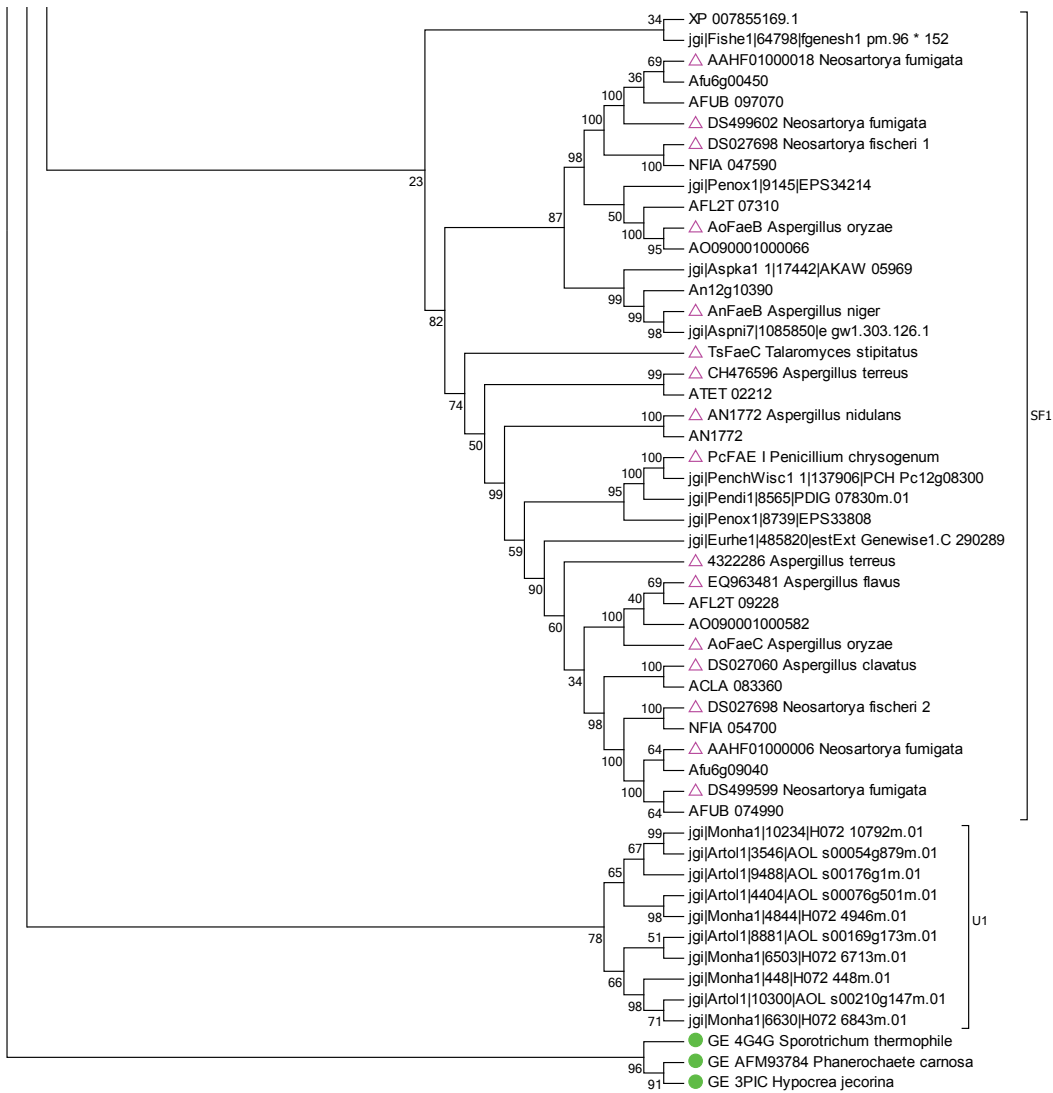

(SF1, U1, Outgroup)

Supplement: Supplementary file 2 — Additional file 2: Figure S1. Phylogenetic tree of the (putative) fungal FAEs. FAEs from previously reported phylogenetic analysis [51] were marked with magenta open triangles for SF1, magenta open rhombuses for SF2-4, magenta filled triangles for SF5, magenta filled rhombuses for SF6, light blue filled squares for SF7, and magenta filled circles for ungrouped ones. AtFAE2 and AtFAE3 are marked with brown filled circles, acetyl xylan esterases are marked with blue filled circles, lipases are marked with yellow filled squares, tannases are marked with purple filled circles, glucuronoyl esterases (as an outgroup) were marked with green filled circles. The same symbols are used in Fig. 2. [file 13068_2016_651_MOESM2_ESM.pdf]
